# Metabolic insights into hypoxia adaptation in adolescent athletes at different altitudes: a cross-sectional study

**DOI:** 10.3389/fmolb.2025.1571103

**Published:** 2025-05-09

**Authors:** Carlos A. R. Sánchez, Daniel Pardo-Rodriguez, Erica Mancera-Soto, Lizeth León, Dailson Paulucio, Angelo D’Alessandro, Caleb G. M. Santos, Edgar Cristancho, Gustavo Monnerat, Diana M. Ramos-Caballero, Mónica P. Cala, Fernando Pompeu

**Affiliations:** ^1^ Federal University of Rio de Janeiro, Biometrics Laboratory (LADEBIO), Rio de Janeiro, Brazil; ^2^ Metabolomics Core Facility, Vice-Presidency for Research, Universidad de los Andes, Bogotá, Colombia; ^3^ Departamento del Movimiento Corporal Humano, Facultad de Medicina, Universidad Nacional de Colombia, Bogotá, Colombia; ^4^ Department of Biochemistry and Molecular Genetics, University of Colorado Denver-Anschutz Medical Campus, Aurora, CO, United States; ^5^ Instituto de Biologia do Exército, Rio de Janeiro, Brazil; ^6^ Departamento de Biología, Facultad de Ciencias, Universidad Nacional de Colombia, Bogotá, Colombia; ^7^ Federal University of Rio de Janeiro, Institute of Biophysics Carlos Chagas Filho, Rio de Janeiro, Brazil; ^8^ Escuela de Medicina y Ciencias de la Salud, Universidad del Rosario, Bogotá, Colombia

**Keywords:** untargeted metabolomics, altitude training, hypoxia, endurance exercise, adolescent

## Abstract

Athletes use hypoxic training methods to enhance their performance under altitude conditions. Comparative studies involving populations from low (500–2,000 m) and moderate (2,000–3,000 m) altitudes offer an opportunity to understand the mechanisms behind adaptations to hypoxia. The present study combined data from metabolomics analysis based on gas- and liquid-chromatography mass spectrometry (GC-MS and LC-MS) to compare plasma profiles from 80 adolescent athletes at moderate- or low altitudes. 161 metabolites were identified, including 84 elevated and 77 decreased in moderate-altitude adolescents compared to their low-altitude counterparts. Pathway analysis revealed that metabolites related to carbohydrates, amino acids, and lipid metabolism differed between groups. Lipid metabolism was significantly altered in moderate-altitude athletes, including pathways such as linolenic and linoleic acid, sphingolipid, and arachidonic acid, as well as processes involving the transfer of acetyl groups into mitochondria and fatty acid biosynthesis. Biomarker analysis looking for signatures of chronic adaptation to moderate altitude identified glycerol and 5-oxoproline metabolites amongst the variables with the strongest sensitivity and specificity. This study demonstrates differences in metabolic profiles between moderate- and low-altitude populations and highlights the potential of these differential metabolites and associated metabolic pathways to provide new insights into the mechanisms of adaptation to moderate altitude.

## 1 Introduction

Biological maturation is a key factor influencing variations in maximum oxygen consumption (VO_2max_) throughout human development (Mancera-Soto et al., 2022a; Mancera-Soto et al., 2022b; Mancera-Soto et al., 2021). Endurance training induces changes in the human body’s structure and function. This leads to concurrent adaptations in various physiological systems, such as the cardiovascular, hematological, and musculoskeletal systems. These adaptations contribute to an enhanced VO_2max_ and improvements in work capacity (Jones and Carter, 2000). For endurance sports, the primary limiting factor for VO_2max_ (Xavier et al., 2019) is the supply of oxygen to active muscles and effective energy management. Since oxygen is carried by hemoglobin, both total hemoglobin mass (Hbt) and blood volume (BV) play crucial roles in determining the oxygen transport capacity in the blood, consequently influencing VO_2max_ (Schmidt and Prommer, 2010). Transition from childhood to adolescence is facilitated by hormonal changes, among which elevation in testosterone levels, especially in males, promotes musculoskeletal growth and erythropoiesis (Roy et al., 2023). Various factors, including altitude and physical training, can alter the oxygen transport capacity (Mancera-Soto et al., 2022a). Exposure to altitude induces specific biological effects in humans, amongst which one of the earliest and most established cascades involves the activation of hypoxia-responsive elements (e.g., hypoxia-inducible factors–HIF-1α and HIF-2α) (Semenza, 2001), the elevation of testosterone and erythropoietin to boost erythropoiesis (Lima et al., 2023). Rapid responses to environmental hypoxia trigger rapid cascades of metabolic (Travis et al., 2016) and cardiorespiratory changes that impact both the transport and utilization of oxygen (Xu et al., 2022), such as the rapid synthesis of allosteric modulators of hemoglobin that favor oxygen off-loading, such as 2,3-bisphosphoglycerate (D’Alessandro et al., 2024) (BPG). Lipid metabolism, like the synthesis of sphingosine 1-phosphate, can further contribute to stabilizing BPG-bound deoxyhemoglobin (Sun et al., 2016; Sun et al., 2017), thus enhancing oxygen off-loading capacity and accelerating oxygen kinetics at a single cell level (Rabcuka et al., 2022). These adaptations are particularly relevant in athletes, with recent findings supporting the positive impact of altitude training on cardiorespiratory fitness parameters in young runners (Saltin et al., 1995). Other studies have demonstrated an increase in blood oxygen-related transport parameters through traditional altitude training methods (Live High: Train High or Live High: Train Low) in trained adolescents (Bahenský et al., 2020; Christoulas et al., 2011). Similar trends have been observed from innovative LLTH (Live Low: Train High) methods, which result favorable hematological alterations in the total number of red blood cells or the total hemoglobin mass and enhance the activity of the transcription factor HIF-1α (Burtscher et al., 2022; Sharma et al., 2023).

As an increasing number of lowland children relocate to high altitudes (Pollard et al., 2001), studying physiological acclimatization to hypoxia and associated conditions becomes more crucial. It is essential to identify the molecular variables that play pivotal roles in this process to understand the mechanisms that counteract the adverse effects of oxygen deficiency. The physiological processes characterizing adaptation to acute and prolonged hypobaric hypoxia exposure at high altitudes encompass ventilation and cardiac function, hematology, and oxygen consumption. These processes aid the body in acclimating to hypoxic conditions. Additionally, hypoxia induces metabolic responses to align ATP synthesis and demand in response to decreased oxidative capacity and increased oxidative stress (Muza et al., 2010; West, 2015; Chicco et al., 2018). Studies investigating metabolic acclimatization in healthy human lowlanders have shown tissue-specific responses in skeletal muscle. These studies suggest a shift away from oxidative processes, including β-oxidation, tricarboxylic acid (TCA) cycle activity, and oxidative phosphorylation, towards increased reliance on carbohydrate metabolism (Horscroft and Murray, 2014; Chicco et al., 2018). Enhanced glycolytic capacity has been proposed due to increased levels of glycolytic intermediates within muscle tissue and the upregulation of glucose transporters and glycolytic enzymes mediated by HIF-1α (Horscroft et al., 2017; Kim et al., 2006; Semenza et al., 1994). In adults, adaptation to hypoxia involves the modulation of related metabolites, as demonstrated in previous research (Liao et al., 2016; Gao et al., 2023). Changes induced by hypoxia include alterations in lipid storage and mobilization, such as decreased circulating high-density lipoproteins, increased triglyceride concentrations, inhibited lipoprotein lipase activity, and suppressed *de novo* lipogenesis (Suzuki et al., 2014). These responses are likely regulated at the transcriptional level through HIF-1/2α and may be influenced by changes in circulating catecholamines, which stimulate lipolysis *via* hormone-sensitive lipase (Siervo et al., 2014). Furthermore, peroxisome proliferator-activated receptor alpha (PPARα), a transcriptional regulator of fatty acid oxidation in the liver, heart, and muscle, has been identified as a key player in hypoxic metabolic remodeling processes (Cole et al., 2016).

Metabolomics is a discipline that studies small molecule metabolites (<2 kDa), which are the end-product of the enzymatic activity of gene products in a biological system (Carneiro et al., 2019). When applied to endurance sports, metabolomics can be a valuable tool for understanding how the body responds to acute, subacute, and chronic exercise and how athletes can optimize their performance and recovery (Jaguri et al., 2023; Nemkov et al., 2023a; Nemkov et al., 2023b; Nemkov et al., 2021; Monnerat et al., 2020). Simultaneously, metabolomics explores adaptive responses to hypoxia, seeking specific metabolites that could be early indicators for conditions like cardiovascular and lung diseases. Metabolomics has contributed to understanding the molecular mechanisms underlying the acute and chronic hypoxic response. This includes identifying altered metabolic pathways under hypoxic conditions and characterizing how these metabolic changes affect cellular functioning (Chang et al., 2019). Overall, most existing studies have aimed to comprehend the cardiorespiratory and hematological changes linked to altitude exposure. However, limited reports investigate moderate-altitude residents’ metabolic effects on adolescent endurance athletes’ metabolic profiles. Recent advances in metabolomic techniques make it possible to uncover plasma metabolic profiles in individuals, providing a better insight into hypoxia adaptation and the opportunity to understand the mechanisms of hypoxia resistance. Understanding around metabolic adaptations to hypoxia could serve to improve multiple aspects of optimizing training strategies, improving altitude acclimatization, and developing personalized approaches to enhance athletic performance and recovery. In this study, we conducted an untargeted metabolomics analysis using chromatographic methods coupled to time-of-flight mass spectrometry approaches to explore the metabolic profiles of trained endurance athletes residing at moderate altitude compared to age and sex-matched counterparts living at low altitude.

## 2 Materials and methods

### 2.1 Participants

The data for this study were collected as part of a larger observational study previously reported (Mancera-Soto et al., 2022a; Mancera-Soto et al., 2022b; Mancera-Soto et al., 2021). The study involved the analysis of 80 adolescent male athletes, with an average age of 15 ± 2 years and VO_2max_ of 56.6 ± 7.7 mL∙min^−1^∙kg^−1^. These athletes specialized in aerobic endurance activities such as inline skating, athletics runners and cycling (Supplementary Table S1) were classified as altitude natives (n = 40), or lowlanders (n = 40) based on their birthplace altitude (Bogotá ∼2,600 m and Tuluá ∼1,000 m). The sexual maturation stage of all participants was determined according to Tanner (Tanner, 1962), and only individuals classified as stage III, IV, or stage V were included in the study. Athletes with a minimum of 2 years of training experience, committing at least 6 h per week to training sessions, conducted at least 3 times per week. Additionally, participants were also required to have lived at their respective altitudes for a minimum of 5 years and not to have changed altitudes for more than 1 week during the preceding year. Participants with recent lower limb musculoskeletal injuries or who reported consuming iron, folic acid, or other supplements that potentially alter the study variables were not allowed to participate. All adolescents and their parents provided informed consent, the experimental procedures adhered to the Declaration of Helsinki (1964), and were approved by the ethical committee of the National University of Colombia at Bogotá (reference: ID 06/2015).

### 2.2 Study design

This cross-sectional study with an independent group design was conducted at four-time points (T1–T4). The evaluation included anthropometric measurements, medical examinations, and ergospirometry measurements following the protocols outlined by Mancera et al., in 2022 (Mancera-Soto et al., 2022a; Mancera-Soto et al., 2022b). Details are included as Supplementary Material. On T1, anthropometric measurements, medical and fitness examinations, and biological maturation assessments were performed. On T2, a cubital venous blood sample of 4 mL of heparinized blood was obtained to determine hemoglobin concentration, hematocrit, reticulocyte count, and metabolomic parameters. In T3, hemoglobin mass was determined using the CO rebreathing method according to the protocols above (Schmidt and Prommer, 2005; Prommer and Schmidt, 2007). On T4, an incremental step test on a cycle ergometer or treadmill was performed to assess VO_2max_. All participants were instructed to abstain from strenuous physical activities (>5METs, defined as the resting metabolic rate) in the 24 h before the evaluations. To avoid methodological errors in the comparison of results, the same equipment was used in the low-altitude and moderate-altitude laboratories, and the same evaluators carried out the tests within a 2-week interval. Cyclists and skaters were tested on a cycle ergometer, and runners were tested on a treadmill. VO_2max_ levels of athletics were adjusted using established correction methods described in the Supplementary Material.

### 2.3 Sample preparation

Plasma metabolites extraction was performed according to the reported methods with some modifications (Pardo-Rodriguez et al., 2023). Briefly, 100 μL of plasma were mixed with 300 μL iced-cold methanol:ethanol (1:1) and vortexed for 2 min (Yang et al., 2013). Then, samples were placed at −20°C for 20 min and centrifuged at 10,000 × *g* for 10 min at 4°C. For GC-MS analysis, 20 μL of the previous extract was evaporated to dryness utilizing a speedvac tool for 90 min at 35°C. Methoxymation was performed by adding 10 μL of methoxyamine hydrochloride (15 mg/mL pyridine) and mixing for 5 min. Methoxymation was carried out at room temperature for 16 h. Samples were silylated at 70°C for 1 h with 10 μL of N, O-Bis(trimethylsilyl) trifluoroacetamide. Finally, 40 µL of heptane (containing 2 ppm of methyl stearate as an internal standard) were added (Fiehn, 2016).

#### 2.3.1 Liquid chromatography-mass spectrometry (LC–MS)

Metabolomic profiling analysis was conducted on an Agilent Technologies 1260 Liquid Chromatography system coupled to a quadrupole time-of-flight (QTOF) mass spectrometer using positive electrospray ionization mode. The chromatography separation of metabolites was performed by an InfinityLab Poroshell 120 EC-C18 (100 × 2.1 mm, 1.9 µm) column at 40°C, with an injection volume of 2 μL and a flow rate of 0.4 mL/min. Mobile phases were 0.1% formic acid in water (A) and 0.1% formic acid in acetonitrile (B). The gradient started at 5% B, and increased to 95% B from 0 to 15 min, and held there for 1 min, then returned to 5% B at 16.1 min and equilibrated there until 20 min. Parameters for mass spectrometry acquisition were as follows: Vcap (V) 3000, gas temp 250°C, drying gas 12 L/min, nebulizer 52 psi, sheat gas temp 370°C, sheat gas flow 11 L/min, fragmentor 175 V, skimmer 65 V and octapole radio frequency voltage of 750 V. Centroid data were collected from 100 to 1,100 m*/z* and a scan rate of 1.02 spectra/s. Mass accuracy was maintained by continuously monitoring internal reference ions, *m/z* 121.0509 (purine) and *m/z* 922.0098 (HP-0921).

#### 2.3.2 Gas chromatography-mass spectrometry (GC–MS)

Samples were analyzed on Agilent Technologies 7890B gas chromatograph coupled to a 7250GC/Q-TOF time-of-flight mass spectrometer. 1 μL of the derivatized sample was injected with a constant gas flow of 0.7 mL/min and a split ratio of 30:1 onto a column HP-5MS (30 m, 0.25 mm, 0.25 µm) (Agilent Technologies). The oven temperature was programmed from 60°C for 1 min, then increased to 325°C with a ramp rate of 10°C/min and held there for 10 min. Mass spectra were recorded at 70 eV in full scan mode with values in the range of 50–600 m*/z*. The temperature of the transfer line to the detector was 280°C, the source filament 230°C, and quadrupole temperature 150°C.

#### 2.3.3 Quality control samples

According to previously reported (Kirwan et al., 2022), quality control and quality assurance were implemented in this study to achieve reliable and consistent data.

#### 2.3.4 Data treatment

Raw files acquired from LC-MS were imported into *Agilent MassHunter Profinder B.10.0* software for feature extraction, using molecular feature extraction and recursive feature extraction algorithms. For GC/MS data, the Agilent Unknowns Analysis B.10.0, the MassProfiler Professional v15, and the Agilent MassHunter Quantitative Analysis B.10.00 software were used for the process of deconvolution, alignment, and integration, respectively. Features that were present in at least 80% of samples within the same group and had a coefficient of variation (CV, %) in QC samples below 20% for LC data (30% for GC data) were selected for statistical analysis.

#### 2.3.5 Statistical analysis of the clinical variables of the study participants

The statistical analyses were conducted using the software JASP (Version 0.17.1; JASP team, 2024). Data normality was assessed using the Shapiro-Wilk test. Variables that met normality assumptions were analyzed using parametric tests. The variables age, height, and training load (hours) did not meet normality assumptions and were compared between groups using the non-parametric Mann-Whitney test. The hematological, ergospirometry, and anthropometry parameters were analyzed using Student’s t-test for independent samples. To evaluate the effects of the sports disciplines in the groups, one-way analysis of variance (ANOVA) for independent measures was run. A Bonferroni *post hoc* test was used for multiple comparisons when statistical differences were found. The dependent variables age (y), height (cm), body mass (kg), fat-free mass (kg), percent fat % and training load (hours) did not meet normality assumptions and were compared between the groups using the non-parametric Kruskal–Wallis test. Effect sizes (ES) were calculated to compare altitude natives and lowlanders groups. Effect sizes of 0.10–0.24, 0.25 to 0.40, and >0.40 were used to indicate small, medium, and large sizes (Cohen, 2013). Using the Spearman correlation method, significant metabolites were used to correlate with physiological, and hematological parameters. Descriptive statistics are reported as mean ± standard deviation (SD). A level of α ≤ 0.05 was considered for statistical significance.

#### 2.3.6 Data processing and analysis

Univariate analysis (UVA) and multivariate analysis (MVA) statistical analyses were performed to identify the molecular features with statistically significant differences between groups. UVA p-values were calculated using nonparametric tests (Mann-Whitney U test) in software MATLAB Version: 9.13.0 (R2022b) Update 2 (Inc, 2022). PCA was employed as an unsupervised method in MVA analysis to evaluate data quality and sample distribution. The molecular features leading to group separation were identified using supervised orthogonal partial least squares regression approach (OPLS-DA) models. The quality and performance of the multivariate OPLS-DA models were evaluated using the *R* ^2^, *Q* ^2^, permutation test, and cross-validation analysis of variance values. MVA analysis was performed using the SIMCA-P+16.0 software (Umetrics). The statistically significant features chosen met at least one of the following requirements: (1) UVA—p-value <0.05 and (2) MVA—variance important in projection (VIP) > 1. The predictive performance of each selected biomarker was assessed through ROC analysis (Supplementary Material).

#### 2.3.7 Metabolites identification

Multiple variables have been employed to annotate relevant characteristics analyzed with liquid chromatography. This includes confirming retention times and the probability of adduct formation, comparing high-resolution masses to database records using the CEU Mass Mediator tool, and developing theoretical formulations based on isotopic distributions. MS/MS data were compared to spectra data obtained from MS-DIAL 4.80, Lipid Annotator software v10.0, and the GNPS server. Manual interpretation of the MS/MS spectra was also carried out. Compound identification through GC analysis was accomplished by comparing the mass spectrum and FAMES retention index to those in the Fiehn GC-MS Metabolomics RTL Library 2013 (Kind et al., 2009). Each platform’s identification levels were assigned based on Blaženović et al.'s Metabolomics Standards Initiative criteria (Blaženović et al., 2018).

#### 2.3.8 ROC curve analysis

ROC (Receiver Operating Characteristic) curve analysis was performed using GraphPad Prism (version 8.0.1, GraphPad Software, San Diego, CA, United States) to evaluate the discriminatory power of the selected metabolites. Only metabolites that met the statistical threshold of adjusted p-value (FDR) < 0.05 and had Level 2 identification confidence (according to the Metabolomics Standards Initiative) were included in the analysis. For each metabolite, the area under the curve (AUC) and its 95% confidence interval were calculated. In addition, the optimal cutoff point was determined based on the maximum Youden index (*J* = sensitivity + specificity−1), which represents the best trade-off between sensitivity and specificity.

## 3 Results

### 3.1 Performance and physiological characteristics

The demographic characteristics of the altitude natives and lowlanders groups are outlined in Table 1. Both groups exhibited similar anthropometric features, except for body fat percentage, indicating distinctions in body composition. There were no significant differences in the weekly training volume between the altitude natives and lowlanders groups. Regarding physiological properties, the maximal heart rate achieved during the maximal exercise test varied between the groups. Relative VO_2max_ and VO_2maxLBM_ obtained in the incremental step test was 4.2 mL∙min [95% CI: (1.75, 6.75); p = 0.001] and 4.3 mL∙min [95% CI: (1.00, 7.60); p = 0.001] higher in altitude natives compared to lowlanders. Moreover, it is worth investigating whether the environmental conditions influence these results during the tests. To facilitate a meaningful comparison between the two groups, we adjusted the VO_2max_ values of the participants from Tuluá to the predicted values for 2,600 m. This adjustment was made using understandings provided by Fulco et al. (1998). According to their regression curves, we observed a 12% decrease in VO_2max_ for participants with values ≤51 mL∙min^−1^∙kg^−1^ and a 20% decrease for participants with values ≥63 mL ∙min^−1^∙kg^−1^. Following this correction, the maximum oxygen consumption altitude (VO_2maxalt_) was found to be 12.1 mL∙min [95% CI: (10.00, 14.32); p = 0.001] higher in the altitude group compared to the lowlanders group. The respiratory quotient was not different between the altitude natives and the lowlanders groups. Significant differences were observed in body fat percentage (*F* = 5.731, p = 0.001) between athletic runners and participants in the inline skating and cycling groups. Additionally, the respiratory quotient (*F* = 19.375, p = 0.001) differed significantly between athletic runners and the cycling group (Supplementary Table S1).

**TABLE 1:** Anthropometric, physiological, and hematological parameters of adolescent athletes participating in the present study.

	Altitude natives Male = 40	Lowlanders Male = 40	p-value	95% CI for effect size	
				Lower	Upper
Anthropometric data					
Age (y)	15.6 ± 1.7 (12–18)	15.2 ± 1.9 (10–18)	0.383	−0.143	0.350
Height (cm)	166.1 ± 7.0 (151–180)	167.4 ± 8.0 (146–181)	0.452	−0.375	0.114
Body mass (kg)	54.9 ± 7.9	54.8 ± 7.8	0.974	−0.431	0.446
BMI (kg∙m^−2^)	19.8 ± 2.0	19.5 ± 1.7	0.413	−0.256	0.623
Fat-free Mass (kg)	48.7 ± 7.2	47.5 ± 7.1	0.442	−0.267	0.611
Percent Fat %	11.2 ± 2.2	13.4 ± 4.3	0.007*	−1.075	−0.177
Performance data					
Training Load (hours per week)	15.3 ± 6.3 (6–33)	15.6 ± 4.9 (6–28)	0.984	−0.172	0.323
HR_max_ (beats∙min^−1^)	192.0 ± 7.0	195.0 ± 8.0	0.022*	−0.966	−0.075
VO_2max_ (mL∙min^−1^∙kg^−1^)	62.8 ± 5.6	58.5 ± 5.6	0.001*	0.301	1.210
VO_2max_ (mL∙min^−1^∙kg^−1^LBM^−1^)	72.2 ± 8.7	67.9 ± 6.0	0.001*	0.131	1.027
VO_2maxalt_ (mL∙min^−1^∙kg^−1^)	62.8 ± 5.6	50.6 ± 4.0	0.001*	1.911	3.087
RQ	1.23 ± 0.10	1.26 ± 0.07	0.163	−0.755	0.127
Hematological data					
Htc (%)	47.3 ± 2.6	44.7 ± 2.8	0.001*	0.480	1.405
[Hb] (g∙dL^−1^)	16.3 ± 1.0	15.1 ± 1.0	0.001*	0.735	1.690
Hbmass (g)	792.3 ± 152.1	742.3 ± 162.7	0.166	−0.127	0.756
EV (mL)	2,295.0 ± 455.4	2,199.0 ± 477.9	0.361	−0.234	0.644
BV (mL)	5,315.6 ± 907.8	5,397.4 ± 1,066.6	0.713	−0.521	0.356
PV (mL)	3,020.6 ± 480.5	3,198.4 ± 618.4	0.115	−0.761	0.121
Hbmass (g∙kg^−1^ LBM)	16.2 ± 1.1	15.5 ± 1.8	0.197	−0.025	0.861
EV (mL∙kg^−1^ LBM)	41.5 ± 3.6	39.6 ± 5.2	0.065	−0.026	0.860
BV (mL∙kg^−1^ LBM)	96.5 ± 6.6	97.6 ± 11.7	0.609	−0.553	0.324
PV (mL∙kg^−1^ LBM)	55.0 ± 4.5	58.0 ± 7.5	0.062	−0.925	−0.035
ARC (10^–−3^ cells∙μL^−1^)	65.1 ± 20.6	48.6 ± 11.2	0.001*	0.522	1.452

Note: Values are presented as mean (SD). Minimum and maximum values. Significance of differences between the groups: *p < 0.05.

Abbreviations: LBM, lean body mass; BMI, body mass index; TL, training load; HR_max,_ maximal heart rate; VO_2max,_ maximal oxygen uptake; RQ, respiratory quotient; Htc, hematocrit; Hb, hemoglobin concentration; Hbmass, hemoglobin mass; ARC, absolute reticulocyte count; BV, blood volume; PV, plasma volume; EV, erythrocyte volume. CI: confidence interval.

### 3.2 Hematological parameters

The outcomes from Table 1 reveal that moderate altitude impacted higher values in nearly all measured hematological parameters related to oxygen transport capacity. Several key hematological parameters exhibited statistical differences. The absolute reticulocyte count was 34% (95% CI: [9.03, 23.81]; p-value = 0.001) higher in altitude natives than lowlanders. Similar differences were observed for hematocrit and hemoglobin concentration (95% CI: [1.36, 3.78]; p = 0.001, 95% CI [0.78, 1.69]; p = 0.001, respectively), all of which showed differences in the altitude natives. However, there were no differences in blood/plasma volumes, hemoglobin mass, and erythrocyte volume between the natives and the lowlanders groups. No statistically significant differences were found between inline skaters, athletic runners, and cyclists in all measured hematological parameters (Supplementary Table S1).

### 3.3 Metabolomics data

Untargeted metabolomics analysis assessed the changed metabolomic profiles linked with individuals from moderate-altitude regions and those from lowland areas in Colombian endurance-trained adolescents. A multiplatform strategy was employed to detect the maximum number of metabolites displaying alterations. Examination of the clusters in the PCA models revealed the grouping of quality control samples across the utilized analytical platforms (Supplementary Figure S1, shown by orange dots), suggesting analytical stability across all platforms utilized.

**FIGURE 1:**
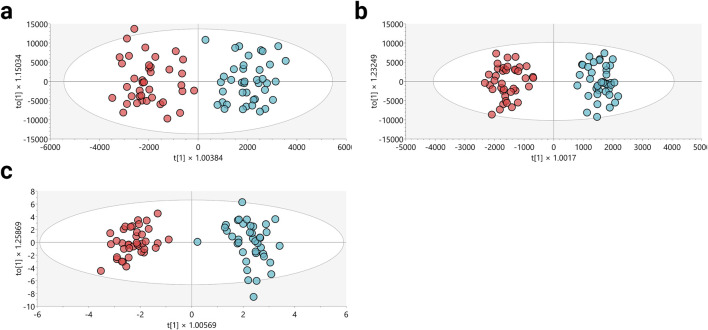
Multivariate models for metabolomic analysis. **(a)** LC-MS(+): *R* ^2^Y: 0.644, *Q* ^2^: 0.659, CV-ANOVA: 1.13^e−12^. **(b)** LC-MS(−): *R* ^2^Y: 0.669, *Q* ^2^: 0.756, CV-ANOVA: 6.92^e−15^. **(c)** GC-MS: *R* ^2^Y: 0.939, *Q* ^2^: 0.614, CV-ANOVA: 1.07^e−11^. Dots in red and blue colors denote samples from altitude natives and the lowlanders group, respectively.

In order to evaluate the potential influence of additional variables on sample clustering, a multivariate PCA approach was also applied to the following variables: type of sport practiced—athletics (ATH), cycling (CYC), or inline skating (SKA); biological maturation status according to Tanner stages (III, IV, and V); hematocrit (Htc%) categorized as <45%, 45%–48%, and >48%; percent fat (%) categorized as <10%, 10%–15%, and >15%; and VO_2_peak (mL∙min^−1^∙kg^−1^) classified as <60, 60–65, and >65 (Supplementary Figure S2). However, the exploration of these variables did not reveal any remarkable clustering trends, which may suggest a limited role as confounding variables in this study. Nevertheless, it is recommended that these variables be further explored and analyzed in future investigations. Following verification of the performance of each analytical platform and the limited influence of additional variables on sample clustering, the supervised OPLS-DA was utilized to enhance the differentiation between the groups (Figures 1a–c). This method aimed to identify the molecular characteristics significantly contributing to the separation of these groups.

The OPLS-DA scoring plot depicted in Figures 1a–c clearly showed distinct clustering between the altitude natives (red dots) and lowlanders (blue dots) across all implemented analytical platforms. Additionally, the metrics *R* ^2^ and *Q* ^2^, evaluating the model’s goodness of fit and predictive ability based on the data, respectively, yielded satisfactory values (*R* ^2^: 0.644–0.939; *Q* ^2^: 0.614–0.756). To ensure the model’s robustness, cross-validation variance (CV-ANOVA) was performed, confirming significant models across all examined platforms (CV-ANOVA <0.05). This observation suggests that the multivariate models avoided overfitting (Eriksson et al., 2008).

**FIGURE 2:**
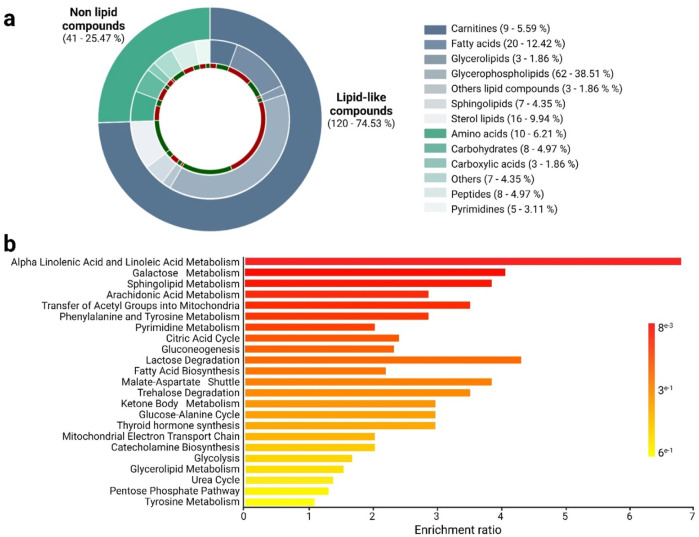
Altered metabolites and biosynthetic pathways among altitude natives and lowlander athletes. **(a)** The chemical classes of altered metabolites between altitude natives and lowlander athletes are shown according to the color code. In green, metabolites are downregulated, and in red, they are upregulated in altitude natives. **(b)** Enrichment analysis of altered pathways in comparing altitude natives and lowlander athletes. The significance of pathway alteration is indicated according to the color scale.

To identify altered metabolites between altitude natives and lowlanders, a combination method that included both UVA with a significance level (*p-*value) below 0.05 and MVA with a VIP threshold over 1 was used. Employing this approach, 161 altered metabolites were found on all the platforms used during the study (Figure 2a). Of these 161 metabolites, around 74.5% (120 metabolites), corresponded to lipid-like compound classes, while 15.5% (41 metabolites) belonged to non-lipid chemical families. Regarding overall trends, it was noted that around 52.2% (84 metabolites) showed an increase, while 47.8% (77 metabolites) exhibited a decrease among moderate-altitude native athletes.

The altered lipid metabolites identified in the study belonged to chemical classes such as carnitines (5.59%), fatty acids (12.42%), glycerolipids (1.86%), glycerophospholipids (38.51%), sphingolipids (4.35%), sterol lipids (9.94%), and others (1.86%). Regarding compound types, no uniform trends were observed across the comparisons, except for the family of sterol lipids, which exhibited a downward trend in altitude natives compared to lowlanders (Figure 2a; Supplementary Table S2). On the other hand, the altered non-lipid compounds identified mainly belonged to chemical classes, including amino acids (6.21%), carbohydrates (4.97%), carboxylic acids (1.86%), peptides (4.97%), pyrimidines (3.11%), and others (4.35%). Notably, trends in certain chemical classes stand out, such as carbohydrates, amino acids, peptides, and carboxylic acids, which were predominantly found to be increased in altitude natives compared to lowlanders (Figure 2a; Supplementary Table S2).

Figure 2b illustrates the global enrichment analysis, revealing dysregulated metabolic pathways between altitude natives and lowlanders. The comparison uncovered multiple dysregulated pathways (highlighted in shades of red), primarily associated with lipid, carbohydrate, and amino acid metabolism. These pathways include lipid metabolism, such as linolenic and linoleic acid, sphingolipid, and arachidonic acid metabolism, as well as the transfer of acetyl groups into mitochondria and fatty acid biosynthesis. Other implicated pathways were found, such as galactose metabolism, phenylalanine and tyrosine metabolism, pyrimidine metabolism, the TCA cycle, and gluconeogenesis. Interestingly, a significant portion of these pathways are related to energy production.

**FIGURE 3:**
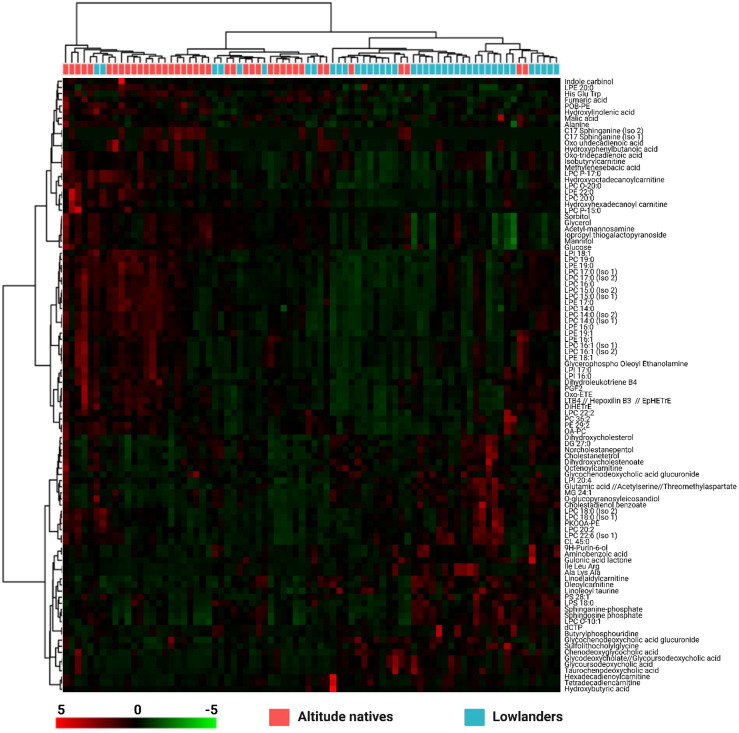
Heatmap of metabolites with statistically significant variation between altitude natives and lowlander athletes. The rows correspond to each altered metabolite identified, the columns correspond to the analyzed samples divided into clades: Altitude natives (red) and lowlanders (blue). The level of variation is indicated on the right side on a color intensity scale representing relative abundance, where red colors denote metabolite increase and green colors denote metabolite decrease.

Given the large number of altered metabolites and aiming to establish more detailed trends among the altered metabolites in the study groups, the set of altered metabolites with the most significant changes (fold change <0.6; >1.4) between the two groups was analyzed using heat maps that allow the visualization of metabolite patterns changing between the groups. Thus, green colors indicate decreased metabolite levels, and red colors indicate increased metabolites (Figure 3).

Interestingly, the heatmap depicted in Figure 3 highlights the robust clustering of samples from the two groups (top clades), suggesting that the metabolites used for its construction enable a clear classification of samples belonging to altitude native (samples in red) and lowlanders (samples in blue) athletes. Regarding the clustering of altered metabolites, three major clades are observed on the left side. The upper clade, comprising metabolites from indole carbinol to glucose, consists of heterogeneous metabolites, including carbohydrates, sphingolipids, carboxylic acid derivatives, and some fatty acids. This clade does not exhibit a prominent trend. However, these metabolites appear to be increased in altitude natives compared to lowlanders. The intermediate clade ranges from LPI 18:1 to OA-PC, consisting predominantly of glycerophospholipids and some fatty acids such as leukotrienes and eicosanoids (important inflammatory mediators), with the lysophospholipids being the most representative. This clade was characterized by being increased in altitude natives compared to lowlanders. Finally, the lower clade encompasses metabolites from dihydroxycholesterol to hydroxybutyric acid. This heterogeneous clade is primarily constituted by some amino acids and peptides, carnitines, other lysophospholipids, and sterol lipids, with the latter class being the most representative. Interestingly, metabolites in this clade exhibited downward trends in altitude natives compared to lowlanders. In summary, two trends can describe most changes found in the clades: lysophospholipids are predominantly increased in altitude natives, while sterol lipids are decreased.

Finally, certain glycerophospholipids showed a high and significant correlation with VO_2max_ data (Supplementary Figure S3). Furthermore, using Spearman correlation, we identified other metabolites that also correlated with VO_2max_ and hematology data. In Supplementary Figure S3, we highlighted all the metabolites that significantly correlated with the physiological and hematological data (Spearman correlation, p-value <0.05, *r* > 0.4 or *r* < −0.4). Besides glycerophospholipids, several other compounds also appear to be highly correlated with VO_2max_ and hematology data.

### 3.4 Potential biomarkers of altitude exposure

**FIGURE 4:**
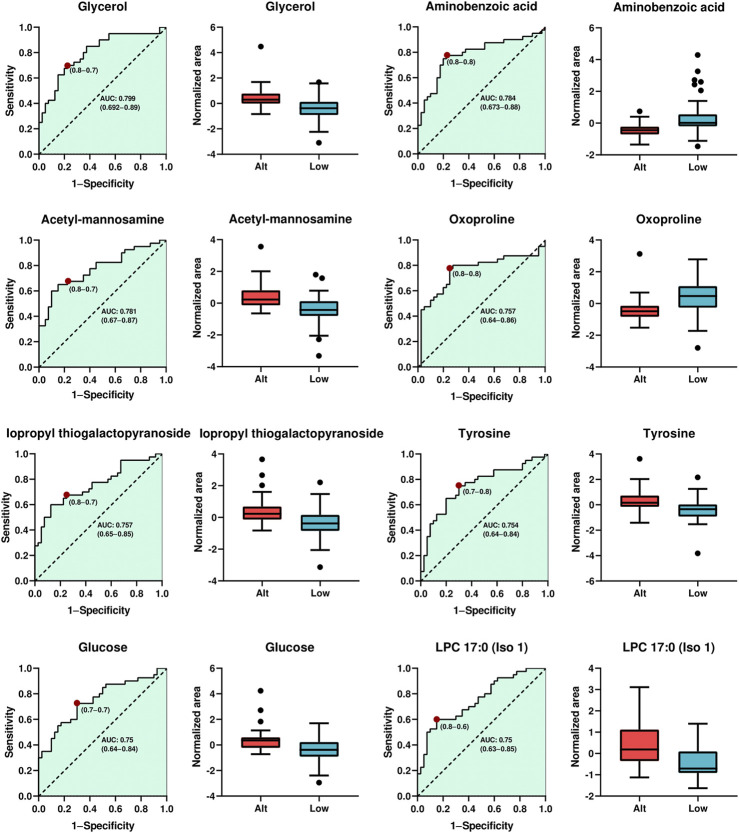
Analysis of the Receiver Operating Characteristics of potential plasma biomarkers for altitude exposure. ROC curve analysis and box plot of metabolites with the highest contribution of separating the studied groups. Boxplot in red and blue colors, representing altitude native and lowlanders athletes, respectively. Data were shown by the median, with the range from minimum to maximum.

Considering their highest levels of identification (ID level 2), fold change, VIP values, and p-values with FDR, a total of eight metabolites (glycerol, aminobenzoic acid, acetyl-mannosamine, oxo-proline, isopropyl thiogalactopyranoside, tyrosine, glucose, LPC17:0) were chosen as potential indicators of altitude exposure in athletes. The predictive performance of each selected biomarker, assessed through ROC analysis, is illustrated in Figure 4. Notably, the findings revealed that glycerol demonstrated the most significant performance, achieving an area under the curve (AUC) value of 0.799 (CI: 0.692–0.89), accompanied by sensitivities and specificities of 0.8 and 0.7, respectively. The remaining seven compounds also exhibit good discriminatory capabilities, with AUC values exceeding 0.75 and sensitivities and specificities greater than 0.6.

## 4 Discussion

**FIGURE 5:**
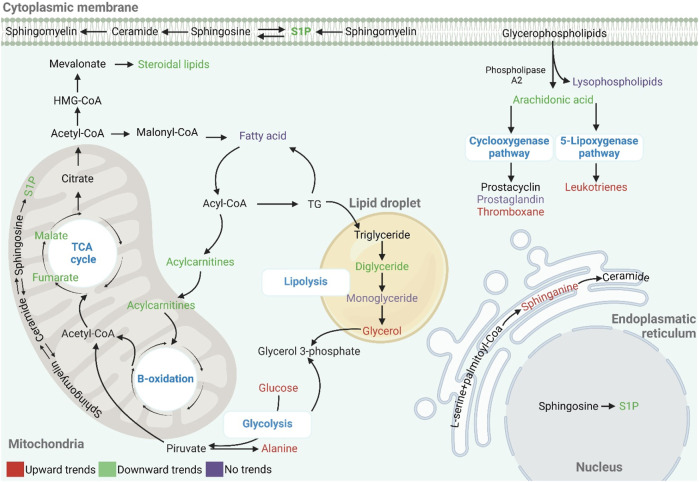
Global metabolic changes in response to hypoxia in adolescent athletes. The metabolites represented in red, green, and purple correspond to increased, decreased, and no trend, respectively.

This research compared the plasma metabolic profiles of adolescents who live and train at moderate altitudes and their counterparts residing at lower altitudes. An untargeted metabolomics approach was employed to identify metabolic profiles and biomarkers associated with chronic adaptation to hypobaric hypoxia. The supervised model analyses discerned distinct metabolic patterns in the two groups, suggesting that chronic residence at moderate altitude is associated with specific metabolic profiles in adolescent endurance athletes. The altered metabolites belong to several classes and include key metabolic pathways related to lipids, including the metabolism of sphingolipids and fatty acids like arachidonic, linolenic, and linoleic acid, fatty acid oxidation, and fatty acid biosynthesis. A variety of processes associated with the TCA cycle, glycolysis, and amino acids are also implicated (Figure 5).

Despite existing studies exploring metabolic shifts during hypoxic exposure in adults at moderate altitudes, the underlying molecular mechanisms in trained adolescents remain relatively unexplored, and the metabolomic nuances of chronic adaptation remain elusive. Recently, Gao et al. (2023) employed metabolomic analysis to study systemic changes resulting from acute hypoxia and found that adenosine, guanosine, inosine, xanthurenic acid, 5-oxo-ETE, raffinose, indole-3-acetic acid and biotin were highly upregulated after exposure at an altitude of 3650 m. Lawler et al. (2019) significant alterations in amino acid metabolism, glycolysis, and purine metabolism, and the time course of these changes is different over 14 days of normobaric, simulated altitude (3,000 m) on the metabolic profile of 10 highly trained middle-distance runners. Liao et al. (2016), analyzed plasma metabolites and revealed significant disruptions in key metabolic pathways at an altitude of 5300 m. These perturbed pathways encompass inflammatory response-related metabolism, energy metabolism, bile acid metabolism, and heme metabolism. According to D’Alessandro et al. (2016), the metabolomic analysis of red blood cells revealed correlations between changes in metabolic and physiological pathways as well as athletic performance parameters following exposure to high altitude (5260 m). The comprehensive metabolomic analysis of muscle biopsies indicated improvements in muscle bioenergetics under physiological hypoxia, showcasing changes in glycolytic intermediates, amino acids, and fatty acids (Chicco et al., 2018). O’Brien et al. (2019) investigated the metabolomic response to progressive exposure to environmental hypoxia in the plasma of 198 healthy individuals before and during an ascent to Everest Base Camp. The study revealed a decrease in isoleucine levels with ascent, along with increasing lactate and decreasing glucose levels, indicating a potential increase in glycolytic rate. All of the findings above highlight the need for a deeper dive into the metabolic mechanisms associated with chronic adaptation to hypobaric hypoxia in adolescents.

In hypoxic high-altitude conditions, the body’s metabolism naturally adjusts to achieve a new dynamic equilibrium. This adaptation results in a metabolic state that differs significantly from that at sea level, reflected in the altered content of various metabolites (Murray, 2009). Metabolism is crucial for bodily function; it maintains metabolic levels within a normal range and is essential for the body’s resistance to the hypoxic conditions of high-moderate altitudes (Chang et al., 2019). In this study, the metabolites analyzed in basal condition could reflect the resting physiological state of adolescent athletes, influenced by their chronic altitude and endurance adaptation. Interestingly, the glycerophospholipids played a crucial role in distinguishing between the groups, with significantly elevated concentrations of certain Lysophosphatidylcholines (LPC) observed in altitude natives (Supplementary Table S2). LPC is primarily generated within cells through the action of phospholipase A2, an enzyme that removes one of the fatty acid groups from phosphatidylcholine (PC) to yield LPC (Law et al., 2019). LPC mediates various cell-signaling pathways within monocytes/macrophages and specific receptors, actively contributing to the inflammatory response (Duong et al., 2004; Kabarowski, 2009; Oestvang and Johansen, 2006). Consistent with our findings, lipidomic studies have shown a notable increase in LPC levels in response to hypoxic exposure. Furthermore, insights from lipid consumption research propose that LPC serves as a more easily accessible nutrient source, supporting cellular proliferation in hypoxic environments (Yu et al., 2014; Kamphorst et al., 2013). On the other hand, several free fatty acids (FFA), including linoleic acid, arachidonic acid, and eicosapentaenoic acid, were lower among altitude natives. Our findings suggest a shift in linoleic acid metabolism, indicating a reconfigured utilization of linoleic acid and arachidonic acid to produce prostaglandins and leukotrienes as molecular byproducts (Supplementary Table S2). In our data, leukotriene B4 and prostaglandin F2 were elevated in the altitude natives group. This could be related to the decrease in arachidonic acid metabolism. Linoleic acid serves as a precursor to arachidonic acid through processes of elongation and unsaturation. Balsinde et al. 2002) have demonstrated that arachidonic acid can undergo metabolism by cyclooxygenases and lipooxygenases, resulting in the formation of various eicosanoids like prostaglandins, thromboxanes, leukotrienes, and lipoxins, all of which play roles in the inflammatory response. We observed an increase in oxo-ETE, a product of arachidonic metabolism, in the altitude natives group (Supplementary Table S2). Gao et al. (2023) have indicated that exposure to hypoxia leads to elevated levels of 5-oxo-ETE in healthy volunteers from lowland regions. This compound acts synergistically with various lipid and peptide mediators, playing a role in regulating inflammation alongside other lipid mediators, chemokines, and cytokines (Erlemann et al., 2007; Grant et al., 2009; Erlemann et al., 2004).

Acetylcarnitines play crucial roles in various cellular energy metabolism pathways and are known to be involved in cellular responses to hypoxia-induced stress (Dambrova et al., 2022; Liao et al., 2016; Gao et al., 2023). Previous studies have shown that acute hypoxia inhibits free fatty acid oxidation, leading to the accumulation of long-chain acylcarnitines (Liao et al., 2016; Bruder and Raff, 2010). Higher urinary acetylcarnitine excretion has been observed in individuals susceptible to acute mountain sickness (Sibomana et al., 2021). Gao et al. (2023) reported an increase in acylcarnitines during hypoxia exposure, which then decreased upon return to low altitude. In contrast, Horscroft et al. (2017) reported lower levels of acylcarnitines in altitude natives compared to lowlanders pre-exposure to high altitude, and these levels remained low during hypoxia exposure. The current study observed a decrease in medium and long-chain acylcarnitine levels in the altitude natives (Supplementary Table S2). We suggest that the carnitine system is essential for moderate altitude adaptation, possibly contributing to elite athletes’ improved endurance performance. The altered behavior of acylcarnitines during altitude exposure reveals multiple nuances that are not yet fully understood. An alternative role for acylcarnitines in the context of high-altitude hypoxia pertains to their role in fueling the Lands cycle, a process of lipid damage repair that involves the enzymes lysophosphatidylcholine acyl-transferase (e.g., LPCAT3) to recyclates lysophospholipids upon phospholipase activation under hypoxic conditions (Wu et al., 2016; Nemkov et al., 2024; Xu et al., 2022).

Sphingosine 1-phosphate (S1P) is a lipid mediator produced through the metabolism of sphingomyelin. S1P is produced through two enzymes—sphingosine kinase 1 (Sphk1) and sphingosine kinase 2 (Sphk2). Previous research has highlighted that extracellular S1P plays a role in various physiological activities, such as immune cell trafficking, hematopoietic stem cell trafficking, vascular integrity, and cell proliferation, achieved by activating its five G-protein-coupled receptors (Mendelson et al., 2014) and also plays a role in protecting the heart against ischemia/reperfusion injury by promoting lymphocyte egress and inhibiting apoptosis. (Karliner, 2013). Sun et al. (2016), showed that Sphk1 activity and S1P levels are induced in mature erythrocytes of lowland volunteers exposed to an altitude of 5,260 m. In the same study, using a mouse model, these authors demonstrated that increased Sphk1 activity in red blood cells (RBCs) contributes to elevated S1P production within RBCs in hypoxic conditions. Similar findings have been reported in the context of *ex vivo* exposure of human or murine RBCs to hypoxia, e.g., in the context of hypoxic storage under blood bank conditions, whereby S1P-dependent promotion of glycolysis came at the expense of the antioxidant potential *via* depression of the NADPH-generating pentose phosphate pathway (Hay et al., 2023). Other authors have reported that (S1P) is involved in the induction of cyclooxygenase-2 (COX-2) and regulates the production of eicosanoids (Yang et al., 2020). Hodun et al. (Hodun et al., 2021), demonstrated that the activation of the inflammatory response plays a role in the reduced S1P levels during prolonged ultra-endurance competition. Interestingly, our results showed low levels of S1P and eicosanoids in the altitude natives, which could play a central role in the responses to intense endurance training (inflammatory reaction) in moderate altitude environments.

Bile acids play an important role in various physiological functions, such as facilitating the absorption of nutrients in the intestines and the secretion of lipids, toxic metabolites, and xenobiotics in bile. Taurochenodeoxycholic acid (TCDCA), a major bile acid, is synthesized by combining taurine and chenodeoxycholic acid (CDCA) in organisms. In our own study, the bile acids TCDCA and CDCA were downregulated in the altitude natives. Previous studies showed the bile acids TCDCA and glycochenodeoxycholate-3-sulfate increased after high altitude exposure (Liao et al., 2016). Additional studies have demonstrated that acute hypobaric hypoxia can lead to substantial damage to the liver and gastrointestinal mucosa (Rong et al., 2009). A recently identified membrane receptor, Gαs protein-coupled receptor (TGR5), responds to bile acid activation (Kawamata et al., 2003). TGR5 is expressed in various tissues, including the gallbladder, spleen, liver, intestine, kidney, skeletal muscle, pancreas, adipocytes, and macrophages. TGR5/cAMP signaling demonstrates anti-inflammatory functions in the liver and intestine by inhibiting nuclear factor NF-κB-mediated inflammatory cytokine production (Wang et al., 2011; El Kasmi et al., 2018). Given that high hypoxic exposure induces pro-inflammatory stimuli with subsequent anti-inflammatory responses, this may elucidate the observed downregulation of TCDCA in the altitude natives group.

The altitude natives exhibited elevated levels of the aromatic amino acid tyrosine. These alterations in amino acid levels suggest metabolic adaptations to fulfill increased energy demands. The decreased ATP production resulting from TCA cycle inhibition triggered by hypobaric hypoxia may prompt the utilization of BCAAs to compensate for energy deficits (Liao et al., 2016). The elevated levels of tyrosine could be attributed to the replenishment of energy reserves necessary to cope with hypoxic stress. Moreover, tyrosine might aid in regulating oxidative stress, immune response, and inflammation, thus providing protection against tissue damage (Chicco et al., 2018; Liu et al., 2017; Shukitt-Hale et al., 1996; Gandhi et al., 2022). The altitude natives also exhibited elevated levels of alanine, a crucial gluconeogenic α-amino acid responsible for delivering carbon derived from amino acid breakdown in peripheral tissues and skeletal muscles. Alanine, a component of all proteins and peptides, serves as a source of energy through the oxidation of pyruvate released by the transamination of alanine. It undergoes conversion into biomolecules like pyruvate, 2-oxoglutarate, and fumarate, which enter the TCA cycle to address ATP depletion and energy requirements, thereby facilitating acclimatization to high altitudes (Gandhi et al., 2022; MA and Ma, 2017, D’Alessandro et al., 2016). On the other hand, isoleucine, a branched-chain amino acid (BCAA), serves as a primary mediator of alanine and glutamine. Our data demonstrated that individuals native to moderate altitude exhibited lower levels of isoleucine than lowlanders. The catabolism of BCAAs initiates in skeletal muscle with the transamination of α-ketoglutarate, producing branched-chain ketoacids, which are further oxidized as succinyl Co-A in the TCA cycle. Additionally, BCAAs play a crucial role in energy metabolism, and their increase indicates impaired energy metabolism due to oxidative stress and compromised mitochondrial respiration (O’Brien et al., 2019; Luo et al., 2012; Pichler Hefti et al., 2013). O’Brien et al. (2019) recently demonstrated that isoleucine decreased in response to progressive exposure to environmental hypoxia in the plasma of healthy individuals. Carboxylic acid metabolism is responsive to hypoxia in mitochondria-endowed cells, whereby electron transport chain uncoupling following limited oxygen availability (e.g., ischemic (Chouchani et al., 2014) or hemorrhagic hypoxia (D’Alessandro et al., 2017), but also exercise (Reddy et al., 2020)) coincides with elevation of succinate and other dicarboxylates that contribute to the stabilization of HIF1α and–along with the stimulation of erythropoiesis–contribute to inflammatory cascades such as the expression of HIF-targets like interleukin 1 beta (Tannahill et al., 2013). In mitochondria-devoid cells, like RBCs, hypoxic metabolic reprogramming also activates non-canonical carboxylate metabolism *via* cytosolic isoforms of TCA cycle enzymes (e.g., IDH1, MDH1, ME1), which contribute to the homeostasis of reducing equivalents through orthogonal pathways than glycolysis and the hexose monophosphate shunt (Nemkov et al., 2017).

In this research, unsupervised biomarker analyses identified relevant metabolites that could indicate chronic adaptation to moderate altitude and cardiorespiratory fitness. These metabolites include glycerol and 5-oxoproline. Benso et al. (2007) showed that hypoxia at altitude can stimulate lipolysis, increasing blood glycerol levels. Other authors have suggested that plasma glycerol measured at rest and after physical activity could potentially serve as a biomarker of cardiorespiratory fitness (Lewis et al., 2010). In our study, the altitude natives group showed less oxo-proline concentrations than lowlanders individuals. Oxoproline, also known as 5-oxoproline or pyroglutamate, is an intermediate in the glutathione metabolism pathway and can accumulate when there is a deficiency in glutathione synthetase (Lu, 2013). In oxoprolinase-deficient mature RBCs, which contribute significantly to the circulating levels of this metabolite, 5-oxoproline is an end product of the gamma-glutamyl cycle (Paglia et al., 2016). Elevated levels of oxoproline are associated with oxidative stress and can serve as a biomarker to assess the redox state of cells and tissues in clinical settings (Sharma et al., 2022). Several studies highlight the role of oxoproline in oxidative stress. One study notes that 5-oxoproline can induce protein oxidation in tissues, marking it a significant oxidative stress indicator. The same study observed that Oplah full-body knock-out mice with elevated oxoproline levels showed increased oxidative stress, cardiac fibrosis, and other symptoms related to heart failure (van der Pol et al., 2018).

Additionally, correlative analyses indicated a linkage between certain glycerophospholipids and VO_2max_ Høeg, Høeg et al. (2020) conducted non-targeted and targeted metabolomic testing on ultramarathon runners’ plasma samples. They found that higher levels of specific phosphatidylcholines were associated with faster finish times in the Western States Endurance Run. This supports the idea that certain glycerophospholipids, like phosphatidylcholine, may be linked to superior aerobic performance and higher VO_2max_ levels. These findings are also consistent with those of Bye et al. (2012), who found that individuals with higher VO_2max_ had lower levels of free choline and higher levels of phosphatidylcholine. This suggests that choline metabolism might be linked to cardiorespiratory fitness, with potential implications for cardiovascular health​ and type 2 diabetes (Newsom et al., 2016; Wallner and Schmitz, 2011; Floegel et al., 2013).

The present study compared the plasma metabolic profiles of athletes trained at moderate and low altitudes to identify the potential metabolic pathways underlying these differences.

The plasma metabolite screening was focused on adolescents trained in endurance sports. Additional studies with larger cohorts are needed, including other populations, such as sedentary adolescents, to fully elucidate the metabolic response to chronic adaptation to moderate altitude. While the current research has provided a comprehensive view of chronic adaptation to altitude and cardiovascular endurance exercise, future studies may consider applying more targeted approaches based on these results to potentially identify metabolites that enhance altitude adaptation processes. Likewise, the study focuses on chronic adaptation but does not account for the possible differences between short-term and long-term metabolic responses to altitude training. Obviously, controlling additional variables such as nutritional intake, sport-specific demands, and training season phase could improve even more future investigations. Moreover, although pre-analytical factors—such as fasting state and sampling time—were controlled, the inclusion of a longitudinal component in future studies would help disentangle the effects of circadian, seasonal, and training-related variation on the metabolome.

## 5 Conclusion

This metabolomic study represents a pioneering exploration of aspects associated with chronic adaptation to moderate altitudes. Using a combined LC-QTOF-MS and GC-QTOF-MS approach, we identified target metabolites that span a wide range of metabolic pathways, shedding light on inflammatory response-related metabolism (such as linoleic acid, arachidonic acid, and phospholipid metabolism), energy metabolism (including glycolysis and fatty acid metabolism), as well as bile acid and sphingolipid metabolism. The metabolites identified in these analyses have the potential to serve as valuable biomarkers for further research into cardiorespiratory fitness and adaptation to moderate altitude in adolescents. This approach provides important insights into the metabolic processes underlying the body’s response to altitude stress and physical training, helping to identify strategies to improve performance and health in these challenging conditions.

## Data Availability

The datasets presented in this study can be found in online repositories. The names of the repository/repositories and accession number(s) can be found in the article/Supplementary Material.
