# Supplementary material for: Metabolic insights into hypoxia adaptation in adolescent athletes at different altitudes: a cross-sectional study
Source: Front Mol Biosci. 2025 May 9;12:1571103. doi: 10.3389/fmolb.2025.1571103 (PMC12098079; doi:10.3389/fmolb.2025.1571103)
Supplement: Supplementary file 1 [file Supplementaryfile1.docx]

**Anthropometric measurements, medical examinations, and ergospirometry measurements.**

Anthropometric measurements were performed by a certified evaluator following standardized protocols, including skinfold assessments at the triceps, subscapular, suprailiac, abdominal, thigh, and medial calf sites, as outlined by (M-JM et al., 2011) Stewart and Marfell-Jones (2011). Estimates of body fat percentage and absolute lean body mass were derived using prediction equations adjusted for age and sex, according to the methodology proposed by (Slaughter et al., 1988). To ensure participant eligibility and safety, a comprehensive medical examination, including resting electrocardiography, was conducted to assess overall health status.

As for the assessment of respiratory and pulmonary gas exchange variables, a breath-by-breath portable gas analyzer (COSMED Model Quark CPET, COSMED^®^, Rome, Italy) was employed. Calibration was performed before each experiment following the manufacturer's instructions. Athletic runners were evaluated on a treadmill (HP Cosmos Quasar, Germany), while skaters and cyclists were assessed on a cycle ergometer (Monark Ergomedic 839, Sweden). Before the commencement of testing, all participants were familiarized with laboratory conditions and the evaluation protocols. A 3-minute warm-up with the initial setting was provided in all tests, and load increments varied among individual groups based on biological differences and the specifics of each sports discipline (Mancera-Soto et al., 2022). The test concluded when the participant could not maintain the number of revolutions (cycle ergometer) or speed (treadmill). Selection of VO_2max_ values occurred when the athlete met at least three of the following criteria during the test: a) VO_2_ plateau ≤ 150 mL·min^-1^, b) respiratory exchange ratio (RER VCO_2_/ VO_2_) ≥ 1.10, c) peak heart rate higher than 90% of the age-predicted, and d) (Borg scale) rating of perceived exertion ≥ 19 (6-20) (Howley et al., 1995). VO₂max levels of runners were adjusted by 7%, following established correction methods (Hermansen and Saltin, 1969). This correction aimed to minimize discrepancies between the testing modalities and ensure comparability across participants from different sport backgrounds.

**Table S1.** Distribution of the different sports included in the study.

|  | INLINE SKATING  N = 10 | ATHLETICS RUNNERS | CYCLING  N = 50 |
| --- | --- | --- | --- |
|  |  | N = 20 |  |
| **ANTHROPOMETRIC DATA** |  |  |  |
| Age (y) | 14.7 ± 2.0 (12-18) | 15.5 ± 1.5 (13-18) | 15.4 ± 1.8 (10-18) |
| Height (cm) | 165.3 ± 6.5 (156-175) | 165.8 ± 7.6 (151-176) | 167.4 ± 7.7 (146-180) |
| Body mass (kg) | 56.0 ± 8.9 (41-70) | 52.8 ± 7.9 (37-63) | 55.4 ± 7.5 (35-70) |
| BMI (kg∙m^-2^) | 20.4 ± 2.6 | 19.1 ± 1.7 | 19.6 ± 1.6 |
| Fat-free Mass (kg) | 48.4 ± 8.1 (35-60) | 47.4 ± 6.9 (33-56) | 48.2 ± 7.1 (28-63) |
| Percent Fat % | 13.5 ± 1.9 (9-15)^#^ | 10.1 ± 2.0 (5-13) | 12.9 ± 3.9 (6-26)^#^ |
| **PERFORMANCE DATA** |  |  |  |
| Training Load (hours per week) | 17.4 ± 7.2 (10-30) | 13.6 ± 4.3 (6-21) | 15.9 ± 5.5 (6-33) |
| HR_max_ (beats∙min^-1^) | 192.0 ± 4.0 | 192.4 ± 6.7 | 194.2 ± 8.0 |
| VO_2max_ (mL∙min^-1^∙kg^-1^) | 58.4 ± 4.2 | 61.2 ± 6.8 | 60.8 ± 5.9 |
| VO_2max_ (mL∙min^-1^∙kg^-1^LBM^-1^) | 69.8 ± 3.8 | 69.3 ± 12.4 | 70.3 ± 5.6 |
| VO_2maxalt_ (mL∙min^-1^∙kg^-1^) | 57.1 ± 5.5 | 59.1 ± 8.7 | 55.5 ± 7.6 |
| RQ | 1.22 ± 0.05 | 1.15 ± 0.07 | 1.27 ± 0.08^#^ |
| **HEMATOLOGICAL DATA** |  |  |  |
| Htc (%) | 47.1 ± 1.7 | 46.0 ± 3.3 | 45.7 ± 3.0 |
| [Hb] (g∙dL^-1^) | 16.3 ± 0.64 | 15.9 ± 1.3 | 15.5 ± 1.1 |
| Hbmass (g) | 816.2 ± 171.7 | 756.9 ± 162.0 | 761.6 ± 157.7 |
| EV (mL) | 2355.7 ± 521.8 | 2194.6 ± 465.3 | 2246.2 ± 461.6 |
| BV (mL) | 5489.5 ± 1163.1 | 5207.3 ± 939.8 | 5389. 5 ± 979.4 |
| PV (mL) | 3133.8 ± 656.0 | 3012.7 ± 520.6 | 3143.2 ± 558.3 |
| Hbmass (g∙kg^-1^ LBM) | 16.7 ± 1.2 | 15.8 ± 1.4 | 15.6 ± 1.6 |
| EV (mL∙kg^-1^ LBM) | 41.8 ± 4.3 | 40.8 ± 3.8 | 40.1 ± 4.9 |
| BV (mL∙kg^-1^ LBM) | 97.6 ± 10.4 | 97.5 ± 7.1 | 96.7 ± 10.3 |
| PV (mL∙kg^-1^ LBM) | 55.8 ± 6.6 | 56.6 ± 5.5 | 56.5 ± 6.7 |
| ARC (10^-3^ cells∙μL^-1^) | 52.8 ± 16.4 | 64.6 ± 24.2 | 54.5 ± 15.3 |

Abbreviations: LBM, lean body mass; BMI, body mass index; TL, training load; HR_max,_ maximal heart rate; VO_2max,_ maximal oxygen uptake; RQ, respiratory quotient; Htc, hematocrit; Hb, hemoglobin concentration; Hbmass, hemoglobin mass; ARC, absolute reticulocyte count; BV, blood volume; PV, plasma volume; EV, erythrocyte volume. CI: confidence interval. Note: Values are presented as mean (SD). Minimum and maximum values. Significance of differences between the groups: ^#^p < 0.05. ^#^Significantly different from athletics runners.

**Table S2.** Altered metabolites between altitude natives vs lowlanders comparisons.

| **Name** | **Formula** | **Molecular Weight** | **RT** | **mz Error  (ppm)** | **Adduct** | ^a^**%CV QC** | **Analytical platform** | ^b^**ID** | **ALTITUDE vs LOWLANDERS** | | | |
| --- | --- | --- | --- | --- | --- | --- | --- | --- | --- | --- | --- | --- |
|  |  |  |  |  |  |  |  |  | ^C^**FC** | ***P value*** | ***P val. FDR*** | **VIP** |
| ***Amino acids*** | | | | | | | | | | | | |
| Glutamic acid // Acetylserine // Threomethylaspartate | C_5_H_9_NO_4_ | 147.0532 | 0.76 | 0 | M-H-H_2_O | 7.28 | LC-QTOF-MS | 3 | 0.71 | 1.58E-08 | 3.40E-07 | 1.20 |
| Oxoproline | C_5_H_7_NO_3_ | 129.0426 | 13.278 |  |  | 4.6 | GC-QTOF-MS | 2 | 0.80 | 5.22E-05 | 6.34E-04 | 2.1 |
| Isoleucine | C_6_H_13_NO_2_ | 131.0946 | 10.295 |  |  | 8.1 | GC-QTOF-MS | 2 | 0.97 | -- | -- | 1.2 |
| Alanine | C_3_H_7_NO_2_ | 89.0477 | 7.502 |  |  | 11.7 | GC-QTOF-MS | 2 | 1.26 | 2.87E-04 | 2.56E-03 | -- |
| Homoserine // Threonine | C_4_H_9_NO_3_ | 119.0582 | 0.63 | 0 | M-H-H_2_O | 6.38 | LC-QTOF-MS | 3 | 1.11 | 3.45E-07 | 3.49E-06 | -- |
| Methyllysine | C_7_H_16_N_2_O_2_ | 160.1212 | 0.63 | 0 | M-H | 5.59 | LC-QTOF-MS | 3 | 1.11 | 4.01E-07 | 3.84E-06 | -- |
| Hydroxyornithine | C_5_H_12_N_2_O_3_ | 148.0848 | 0.63 | 3 | M-H-H_2_O | 5.59 | LC-QTOF-MS | 3 | 1.10 | 2.22E-06 | 2.01E-05 | -- |
| Aminoisobutyric acid | C_4_H_9_NO_2_ | 103.0633 | 12.406 |  |  | 11.3 | GC-QTOF-MS | 2 | 1.15 | 1.86E-23 | 2.16E-21 | -- |
| Tyrosine | C_9_H_11_NO_3_ | 181.0739 | 17.853 |  |  | 3.9 | GC-QTOF-MS | 2 | 1.17 | 6.83E-05 | 7.21E-04 | 1.9 |
| Iminodiacetic Acid | C_4_H_7_NO_4_ | 133.0375 | 12.505 |  |  | 8.8 | GC-QTOF-MS | 2 | 1.13 | 5.92E-03 | 4.17E-02 | 1.5 |
| ***Benzenoids*** | | | | | | | | | | | | |
| Aminobenzoic acid | C_7_H_7_NO_2_ | 137.0477 | 16.502 |  |  | 11.6 | GC-QTOF-MS | 2 | 0.58 | 6.94E-06 | 1.15E-04 | -- |
| ***Carbohydrate*** | | | | | | | | | | | | |
| Galactitol | C_6_H_14_O_6_ | 182.079 | 17.68 | -- |  | 6.3 | GC-QTOF-MS | 2 | 0.89 | 4.54E-02 | -- | 1.3 |
| Ribose | C_5_H_10_O_5_ | 150.0528 | 15.018 | -- |  | 7.3 | GC-QTOF-MS | 2 | 0.86 | 4.87E-02 | -- | -- |
| Glycerol | C_3_H_8_O_3_ | 92.0473 | 17.85 | -- |  | 3.1 | GC-QTOF-MS | 2 | 1.32 | 1.75E-06 | 6.75E-05 | 2.4 |
| Acetyl-mannosamine | C_8_H_15_NO_6_ | 221.0899 | 18.902 | -- |  | 2.6 | GC-QTOF-MS | 2 | 1.28 | 1.33E-05 | 1.93E-04 | 2.4 |
| Iopropyl thiogalactopyranoside | C_9_H_18_O_5_S | 238.0875 | 18.901 | -- |  | 3.5 | GC-QTOF-MS | 2 | 1.29 | 5.46E-05 | 6.34E-04 | 2.2 |
| Glucose | C₆H₁₂O₆ | 180.0634 | 17.535 | -- |  | 3.3 | GC-QTOF-MS | 2 | 1.30 | 7.80E-05 | 7.54E-04 | 1.7 |
| Mannitol | C_6_H_14_O_6_ | 182.079 | 17.659 | -- |  | 1.4 | GC-QTOF-MS | 2 | 1.21 | 2.43E-07 | 1.41E-05 | -- |
| Sorbitol | C_6_H_14_O_6_ | 182.079 | 17.66 |  |  | 2.4 | GC-QTOF-MS | 2 | 1.26 | 6.26E-06 | 1.15E-04 | -- |
| ***Carnitines*** | | | | | | | | | | | | |
| Octenoylcarnitine | C_15_H_27_NO_4_ | 285.194 | 5.82 | 5 | M+H | 4.8 | LC-QTOF-MS | 3 | 0.70 | 2.01E-03 | 3.49E-03 | -- |
| Decatrienoylcarnitine | C_17_H_27_NO_4_ | 309.194 | 6.77 | 4 | M+H | 6.6 | LC-QTOF-MS | 2 | 0.82 | 6.48E-03 | 9.67E-03 | -- |
| Tetradecadiencarnitine | C_21_H_37_NO_4_ | 367.2723 | 9.49 | 5 | M+H | 5.8 | LC-QTOF-MS | 2 | 0.65 | 1.00E-02 | 1.35E-02 | -- |
| Hexadecadienoylcarnitine | C_23_H_41_NO_4_ | 395.3036 | 10.55 | 5 | M+H | 9.1 | LC-QTOF-MS | 3 | 0.61 | 2.80E-02 | 3.35E-02 | -- |
| Oleoylcarnitine | C_25_H_47_NO_4_ | 425.3505 | 12.37 | 5 | M+H | 6.1 | LC-QTOF-MS | 3 | 0.72 | 1.11E-03 | 2.19E-03 | 1.19 |
| Linoelaidylcarnitine | C_25_H_45_NO_4_ | 423.3349 | 11.62 | 5 | M+H | 3.8 | LC-QTOF-MS | 3 | 0.71 | 1.23E-04 | 4.03E-04 | 1.14 |
| Hydroxyoctadecanoylcarnitine | C_25_H_49_NO_5_ | 443.3611 | 12.93 | 3 | M+Cl | 11.55 | LC-QTOF-MS | 3 | 1.33 | 1.65E-03 | 3.55E-03 | -- |
| Hydroxyhexadecanoyl carnitine | C_23_H_45_NO_5_ | 415.3298 | 12.87 | 3 | M+Cl | 7.72 | LC-QTOF-MS | 3 | 1.47 | 7.33E-05 | 3.02E-04 | -- |
| Isobutyrylcarnitine | C_11_H_22_NO_4_ | 232.1543 | 9.57 | 2 | M+Cl | 5.01 | LC-QTOF-MS | 3 | 1.26 | 3.40E-04 | 1.06E-03 | -- |
| ***Cardiolipins*** | | | | | | | | | | | | |
| CL 45:0 | C_54_H_104_O_17_P_2_ | 1086.6749 | 11.48 | 7 | M+H | 3.5 | LC-QTOF-MS | 3 | 0.75 | 1.02E-04 | 3.61E-04 | 1.02 |
| ***Carboxylic acids*** | | | | | | | | | | | | |
| Hydroxyphenylbutanoic acid | C_10_H_12_O_3_ | 180.0786 | 7.88 | 4 | M+H | 5.53 | LC-QTOF-MS | 3 | 1.77 | 1.56E-02 | 1.95E-02 | -- |
| Fumaric acid | C_4_H_4_O_4_ | 116.0109 | 10.94 | -- | -- | 10.4 | GC-QTOF-MS | 2 | 1.36 | 1.17E-03 | 9.68E-03 | 1.6 |
| Malic acid | C_4_H_6_O_5_ | 134.0215 | 12.697 | -- | -- | 6.0 | GC-QTOF-MS | 2 | 1.27 | 1.58E-02 | -- | -- |
| ***Fatty acids*** | | | | | | | | | | | | |
| Linoleic acid | C_18_H_32_O_2_ | 280.2402 | 12.8 | 5 | M+H | 2.9 | LC/GC-QTOF-MS | 2 | 0.84 | 3.75E-03 | 5.91E-03 | -- |
| HETE | C_20_H_32_O_3_ | 320.2351 | 12.8 | 1 | M-H | 5.97 | LC-QTOF-MS | 2 | 0.86 | 2.33E-02 | 3.68E-02 | -- |
| O-glucopyranosyleicosandiol | C_26_H_52_O_7_ | 476.3713 | 14.5 | 1 | M-H-H_2_O | 6.80 | LC-QTOF-MS | 3 | 0.57 | 3.33E-08 | 5.73E-07 | -- |
| Eicosapentaenoic acid (Iso 1) | C_20_H_30_O_2_ | 302.2246 | 12.24 | 5 | M+H | 4.62 | LC-QTOF-MS | 2 | 0.85 | 3.16E-02 | 3.63E-02 | -- |
| Eicosapentaenoic acid (Iso 2) | C_20_H_30_O_2_ | 302.2246 | 12.8 | 4 | M+H | 2.0 | LC-QTOF-MS | 3 | 0.83 | 7.09E-03 | 1.01E-02 | -- |
| Linoleoyl taurine | C_20_H_37_NO_4_S | 387.2443 | 12.22 | 2 | M-H | 5.33 | LC-QTOF-MS | 3 | 0.61 | 4.66E-05 | 2.11E-04 | -- |
| Hydroxypalmitic acid | C_16_H_32_O_3_ | 272.2351 | 14.57 | 1 | M-H | 6.08 | LC-QTOF-MS | 3 | 0.83 | 1.65E-03 | 3.55E-03 | 1.03 |
| Arachidonic acid | C_20_H_32_O_2_ | 304.2402 | 15.32 | 1 | M-H | 6.49 | LC-QTOF-MS | 2 | 0.82 | 5.09E-03 | 1.12E-02 | 2.18 |
| Hydroxylinolenic acid | C_18_H_30_O_3_ | 294.2195 | 12.36 | 0 | M-H | 4.33 | LC-QTOF-MS | 3 | 1.33 | 5.93E-03 | 1.10E-02 | 2.62 |
| Oxo-tridecadienoic acid | C_13_H_20_O_3_ | 224.1412 | 9.57 | 1 | M-H | 3.77 | LC-QTOF-MS | 3 | 1.27 | 1.05E-04 | 4.20E-04 | 1.05 |
| Methylenesebacic acid | C_12_H_18_O_4_ | 226.1205 | 9.57 | 3 | M+H-H_2_O | 3.6 | LC-QTOF-MS | 3 | 1.23 | 3.52E-04 | 9.96E-04 | -- |
| Oxo-ETE | C_20_H_30_O_3_ | 318.2195 | 10.63 | 4 | M+H | 4.2 | LC-QTOF-MS | 3 | 1.45 | 9.49E-04 | 1.97E-03 | -- |
| Oxo undecadienoic acid | C_11_H_16_O_3_ | 196.1099 | 7.88 | 1 | M-H | 7.05 | LC-QTOF-MS | 3 | 1.67 | 2.40E-02 | 3.76E-02 | -- |
| Dihydroleukotriene B4 | C_20_H_34_O_4_ | 338.2457 | 11.06 | 2 | M-H | 12.05 | LC-QTOF-MS | 3 | 1.51 | 6.20E-05 | 2.67E-04 | -- |
| LTB4 // Hepoxilin B3 // EpHETrE | C_20_H_32_O_4_ | 336.2301 | 10.63 | 2 | M-H | 10.26 | LC-QTOF-MS | 3 | 1.89 | 3.81E-06 | 2.26E-05 | -- |
| Oxo-ODE // HOTE | C_18_H_30_O_3_ | 294.2195 | 11.8 | 2 | M-H | 8.61 | LC-QTOF-MS | 3 | 1.19 | 2.70E-02 | 4.07E-02 | -- |
| HETrE | C_20_H_34_O_3_ | 322.2508 | 12.94 | 2 | M-H | 6.34 | LC-QTOF-MS | 3 | 1.18 | 1.67E-02 | 2.76E-02 | -- |
| DiHETrE | C_20_H_34_O_4_ | 338.2457 | 10.13 | 1 | M-H | 5.74 | LC-QTOF-MS | 3 | 1.34 | 9.40E-04 | 2.34E-03 | 1.07 |
| DiHETE | C_20_H_32_O_4_ | 336.2301 | 10.81 | 1 | M-H | 9.05 | LC-QTOF-MS | 3 | 1.18 | 4.45E-02 | -- | -- |
| Adipamide | C_6_H_12_N_2_O_2_ | 144.09 | 0.63 | 2 | M-H | 5.73 | LC-QTOF-MS | 3 | 1.11 | 1.16E-06 | 1.33E-05 | -- |
| ***Glycerolipids*** | | | | | | | | | | | | |
| MG 24:1 | C_27_H_52_O_4_ | 440.3866 | 14.87 | 3 | M+HCOOH-H | 8.43 | LC-QTOF-MS | 3 | 0.59 | 4.09E-08 | 6.39E-07 | -- |
| DG 27:0 | C_30_H_58_O_5_ | 498.4284 | 14.07 | 10 | M+Na | 15.5 | LC-QTOF-MS | 3 | 0.65 | 5.30E-05 | 2.81E-04 | -- |
| MG 16:0 | C_19_H_38_O_4_ | 330.277 | 11.66 | 5 | M+H-H_2_O | 5.38 | LC/GC-QTOF-MS | 3 | 1.13 | 3.58E-02 | 4.28E-02 | -- |
| ***Glycerophospholipids*** | | | | | | | | | | | | |
| PHOOA-PC | C_32_H_60_NO_10_P | 649.3955 | 12.57 | 4 | M+H | 4.65 | LC-QTOF-MS | 2 | 0.80 | 4.12E-02 | 4.54E-02 | -- |
| LPC O-10:1 | C_18_H_38_NO_6_P | 395.2437 | 9.59 | 0 | M-H-H_2_O | 4.17 | LC-QTOF-MS | 3 | 0.70 | 2.79E-07 | 3.31E-06 | -- |
| LPC 18:0 (Iso 1) | C_26_H_52_NO_8_P | 537.3431 | 9.25 | 8 | M+Na | 7.64 | LC-QTOF-MS | 2 | 0.80 | 1.38E-02 | 1.75E-02 | -- |
| LPC 18:0 (Iso 2) | C_26_H_52_NO_8_P | 537.3431 | 9.5 | 8 | M+Na | 3.8 | LC-QTOF-MS | 2 | 0.79 | 1.17E-02 | 1.56E-02 | -- |
| LPC 18:2 | C_26_H_50_NO_7_P | 519.3325 | 11.24 | 5 | M+H | 3.3 | LC-QTOF-MS | 2 | 0.83 | 1.55E-03 | 2.93E-03 | 3.01 |
| LPC 20:2 | C_28_H_52_NO_8_P | 561.3431 | 9.37 | 8 | M+Na | 5.4 | LC-QTOF-MS | 2 | 0.77 | 1.20E-02 | 1.56E-02 | -- |
| LPC 20:4 (Iso 1) | C_28_H_50_NO_7_P | 543.3325 | 11.3 | 4 | M+H | 2.26 | LC-QTOF-MS | 2 | 0.85 | 6.77E-03 | 9.75E-03 | 1.13 |
| LPC 20:4 (Iso 2) | C_28_H_50_NO_7_P | 543.3325 | 11.51 | 5 | M+H | 1.6 | LC-QTOF-MS | 2 | 0.83 | 3.37E-04 | 9.87E-04 | 3.21 |
| LPC 22:6 (Iso 1) | C_30_H_50_NO_7_P | 567.3325 | 11.29 | 4 | M+H | 4.39 | LC-QTOF-MS | 2 | 0.79 | 1.66E-03 | 3.00E-03 | -- |
| LPC 22:6 (Iso 2) | C_30_H_50_NO_7_P | 567.3325 | 11.47 | 4 | M+H | 1.7 | LC-QTOF-MS | 2 | 0.83 | 6.20E-03 | 9.41E-03 | 1.19 |
| LPC 20:4 | C_28_H_50_NO_7_P | 543.3325 | 11.52 | 2 | M+Cl | 13.88 | LC-QTOF-MS | 3 | 0.86 | 1.49E-03 | 3.36E-03 | -- |
| PC 36:2 | C_44_H_84_NO_8_P | 785.5935 | 14.38 | 1 | M+Na | 14.3 | LC-QTOF-MS | 3 | 0.74 | -- | -- | 2.18 |
| OA-PC | C_35_H_66_NO_10_P | 691.4424 | 14.34 | 5 | M+H | 15.21 | LC-QTOF-MS | 2 | 1.20 | 3.35E-02 | 3.79E-02 | -- |
| LPC 16:0 | C_24_H_50_NO_7_P | 495.3325 | 12.75 | 2 | M-H | 4.87 | LC-QTOF-MS | 3 | 1.25 | 2.76E-04 | 9.11E-04 | 2.33 |
| LPC 14:0 | C_22_H_46_NO_7_P | 467.3012 | 11 | 2 | M-H | 5.55 | LC-QTOF-MS | 3 | 1.40 | 1.42E-05 | 9.02E-05 | -- |
| LPC 15:1 | C_23_H_46_NO_7_P | 479.3012 | 11.15 | 2 | M-H | 7.10 | LC-QTOF-MS | 3 | 1.18 | 1.53E-02 | 2.55E-02 | -- |
| LPC 22:2 | C_30_H_58_NO_7_P | 575.3951 | 13.89 | 3 | M+HCOOH-H | 6.11 | LC-QTOF-MS | 3 | 1.22 | 1.35E-02 | 2.30E-02 | -- |
| LPC 19:0 | C_27_H_56_NO_7_P | 537.3794 | 14.32 | 2 | M+FA-H | 6.10 | LC-QTOF-MS | 3 | 1.20 | 3.94E-03 | 7.62E-03 | -- |
| LPC O-19:0 | C_27_H_56_NO_7_P | 537.3794 | 14.32 | 5 | M+H | 5.8 | LC-QTOF-MS | 2 | 1.16 | 2.49E-02 | 3.07E-02 | -- |
| LPC P-17:0 | C_25_H_52_NO_6_P | 493.3532 | 12.99 | 4 | M+H | 4.8 | LC-QTOF-MS | 2 | 1.38 | 7.36E-04 | 1.65E-03 | -- |
| LPC 14:0 (Iso 1) | C_22_H_46_NO_7_P | 467.3012 | 10.31 | 5 | M+H | 3.36 | LC-QTOF-MS | 2 | 1.38 | 3.75E-04 | 9.96E-04 | -- |
| LPC 14:0 (Iso 2) | C_22_H_46_NO_7_P | 467.3012 | 10.57 | 5 | M+H | 2.5 | LC-QTOF-MS | 2 | 1.42 | 7.02E-04 | 1.61E-03 | 2.08 |
| LPC P-15:0 | C_23_H_48_NO_6_P | 465.3219 | 11.44 | 5 | M+H | 9.9 | LC-QTOF-MS | 3 | 3.44 | 4.07E-04 | 1.02E-03 | -- |
| LPC 16:1 (Iso 2) | C_24_H_48_NO_7_P | 493.3168 | 10.74 | 5 | M+H | 4.18 | LC-QTOF-MS | 3 | 1.36 | 3.52E-03 | 5.75E-03 | -- |
| LPC 16:1 (Iso 1) | C_24_H_48_NO_7_P | 493.3168 | 10.99 | 5 | M+H | 1.8 | LC-QTOF-MS | 2 | 1.38 | 3.68E-03 | 5.91E-03 | 2.04 |
| LPC 15:0 (Iso 1) | C_23_H_48_NO_7_P | 481.3168 | 11 | 5 | M+H | 3.53 | LC-QTOF-MS | 2 | 1.42 | 7.74E-05 | 3.04E-04 | -- |
| LPC 15:0 (Iso 2) | C_23_H_48_NO_7_P | 481.3168 | 11.27 | 5 | M+H | 2.0 | LC-QTOF-MS | 3 | 1.40 | 1.10E-04 | 3.73E-04 | 1.96 |
| LPC 17:0 (Iso 1) | C_25_H_52_NO_7_P | 509.3481 | 12.51 | 5 | M+H | 3.4 | LC-QTOF-MS | 2 | 1.38 | 3.38E-05 | 2.23E-04 | 1.29 |
| LPC 17:0 (Iso 2) | C_25_H_52_NO_7_P | 509.3481 | 12.75 | 6 | M+H | 3.79 | LC-QTOF-MS | 2 | 1.27 | 1.93E-03 | 3.41E-03 | 2.09 |
| LPC O-20:0 | C_28_H_60_NO_6_P | 537.4158 | 15.61 | 5 | M+H-M+HCOOH-H | 19.59 | LC-QTOF-MS | 2 | 1.21 | 1.01E-02 | 1.53E-02 | -- |
| LPC O-17:0 | C_25_H_54_NO_6_P | 495.3689 | 13.13 | 5 | M+H | 6.49 | LC-QTOF-MS | 2 | 1.17 | 4.34E-02 | -- | -- |
| LPC 20:0 | C_28_H_58_NO_7_P | 551.3951 | 13.92 | 4 | M+H | 5.2 | LC-QTOF-MS | 2 | 1.67 | 4.06E-04 | 1.02E-03 | -- |
| LPC 17:1 | C_25_H_50_NO_7_P | 507.3325 | 11.71 | 8 | M+H M-H | 1.7 | LC-QTOF-MS | 2 | 1.19 | 1.15E-03 | 2.22E-03 | -- |
| LPE 20:2 | C_25_H_48_NO_7_P | 505.3168 | 11.24 | 2 | M-H | 4.38 | LC-QTOF-MS | 3 | 0.84 | 8.14E-04 | 2.12E-03 | 3.66 |
| LPE 22:4 | C_27_H_48_NO_7_P | 529.3168 | 11.51 | 2 | M-H | 3.79 | LC-QTOF-MS | 3 | 0.83 | 1.59E-04 | 5.83E-04 | 3.46 |
| LPE 24:6 | C_29_H_48_NO_7_P | 553.3168 | 11.47 | 2 | M-H | 3.22 | LC-QTOF-MS | 3 | 0.83 | 4.21E-03 | 8.04E-03 | 1.27 |
| LPE 22:2 | C_27_H_52_NO_7_P | 533.3481 | 12.79 | 3 | M-H | 6.73 | LC-QTOF-MS | 3 | 0.86 | 5.90E-03 | 1.10E-02 | -- |
| LPE 26:0 | C_31_H_62_NO_8_P | 607.4213 | 14.59 | 2 | M-H | 8.53 | LC-QTOF-MS | 3 | 0.85 | 8.17E-03 | 1.48E-02 | -- |
| OOB-PE | C_27_H_50_NO_9_P | 563.3223 | 9.51 | 1 | M-H-H_2_O | 7.56 | LC-QTOF-MS | 3 | 0.81 | 9.25E-03 | 1.64E-02 | -- |
| PKOOA-PE | C_29_H_52_NO_10_P | 605.3329 | 9.24 | 2 | M-H | 6.59 | LC-QTOF-MS | 3 | 0.77 | 7.10E-04 | 1.91E-03 | -- |
| POB-PE | C_25_H_48_NO_9_P | 537.3067 | 9.47 | 2 | M-H | 12.20 | LC-QTOF-MS | 3 | 1.37 | 2.45E-02 | 3.79E-02 | -- |
| Glycerophospho Oleoyl Ethanolamine | C_23_H_46_NO_7_P | 479.3012 | 10.99 | 2 | M-H | 4.50 | LC-QTOF-MS | 3 | 1.35 | 9.75E-04 | 2.39E-03 | 2.57 |
| LPE 16:0 | C_21_H_44_NO_7_P | 453.2855 | 10.57 | 2 | M-H | 2.83 | LC-QTOF-MS | 3 | 1.37 | 2.70E-04 | 9.11E-04 | 2.39 |
| LPE 16:1 | C_21_H_42_NO_7_P | 451.2699 | 10.92 | 2 | M-H | 7.22 | LC-QTOF-MS | 3 | 1.84 | 1.28E-05 | 6.86E-05 | -- |
| LPE 17:0 | C_22_H_46_NO_7_P | 467.3012 | 11.27 | 2 | M-H | 5.21 | LC-QTOF-MS | 3 | 1.35 | 3.62E-05 | 1.89E-04 | 2.24 |
| LPE 18:1 | C_23_H_46_NO_7_P | 479.3012 | 10.74 | 2 | M-H | 5.42 | LC-QTOF-MS | 3 | 1.32 | 1.49E-03 | 3.36E-03 | -- |
| LPE 19:0 | C_24_H_50_NO_7_P | 495.3325 | 12.59 | 3 | M-H | 5.22 | LC-QTOF-MS | 3 | 1.39 | 5.52E-06 | 3.16E-05 | 2.53 |
| LPE 19:1 | C_24_H_48_NO_7_P | 493.3168 | 11.67 | 3 | M-H | 10.11 | LC-QTOF-MS | 3 | 1.43 | 1.03E-06 | 1.27E-05 | 1.13 |
| LPE 20:0 | C_25_H_52_NO_7_P | 509.3481 | 12.3 | 3 | M+FA-H | 9.05 | LC-QTOF-MS | 3 | 1.36 | 1.08E-03 | 2.61E-03 | -- |
| LPE 22:0 | C_27_H_56_NO_7_P | 537.3794 | 13.91 | 3 | M-H | 7.07 | LC-QTOF-MS | 3 | 1.71 | 2.04E-04 | 7.02E-04 | -- |
| LPE O-20:0 | C_25_H_54_NO_6_P | 495.3689 | 13.93 | 2 | M-H | 6.19 | LC-QTOF-MS | 3 | 1.16 | 3.40E-02 | -- | -- |
| PE 29:2 | C_34_H_64_NO_8_P | 645.437 | 13.88 | 3 | M+HCOOH-H | 15.02 | LC-QTOF-MS | 3 | 1.20 | 2.50E-02 | 3.84E-02 | -- |
| LPI 16:0 | C_25_H_49_O_12_P | 572.2962 | 11.82 | 2 | M-H M+H-H_2_O | 5.92 | LC-QTOF-MS | 2 | 1.25 | 3.27E-02 | 4.80E-02 | 1.97 |
| LPI 17:0 | C_26_H_51_O_12_P | 586.3118 | 12.75 | 2 | M-H | 7.90 | LC-QTOF-MS | 3 | 1.52 | 3.73E-05 | 1.78E-04 | -- |
| LPI 18:1 | C_27_H_51_O_12_P | 598.3118 | 12.53 | 2 | M-H | 9.70 | LC-QTOF-MS | 2 | 1.32 | 2.84E-03 | 5.69E-03 | -- |
| LPI 18:2 | C_27_H_49_O_12_P | 596.2962 | 11.26 | 2 | M-H | 6.70 | LC-QTOF-MS | 2 | 0.82 | 5.69E-03 | 1.08E-02 | -- |
| LPI 20:4 | C_29_H_49_O_12_P | 620.2962 | 11.32 | 2 | M-H | 6.48 | LC-QTOF-MS | 2 | 0.79 | 5.97E-05 | 2.63E-04 | 1.35 |
| PIM1 37:2 | C_52_H_95_O_18_P | 1038.6256 | 11.46 | 2 | M-H-H_2_O | 7.35 | LC-QTOF-MS | 3 | 0.88 | 3.24E-03 | 6.40E-03 | -- |
| LPS 18:0 | C_24_H_48_NO_9_P | 525.3067 | 13.6 | 2 | M-H | 9.24 | LC-QTOF-MS | 2 | 0.59 | 3.91E-04 | 1.18E-03 | -- |
| PS 28:1 | C_34_H_64_NO_10_P | 677.4268 | 14.04 | 4 | M+H | 7.4 | LC-QTOF-MS | 3 | 0.73 | 3.01E-03 | 5.11E-03 | -- |
| PS 29:1 | C_35_H_66_NO_10_P | 691.4424 | 14.34 | 2 | M-H | 8.89 | LC-QTOF-MS | 3 | 1.19 | 3.73E-02 | -- | -- |
| LPS 26:0 | C_32_H_62_NO_10_P | 651.4111 | 13.07 | 2 | M-H | 7.54 | LC-QTOF-MS | 3 | 1.15 | 4.79E-02 | -- | -- |
| ***Hydroxy acids*** | | | | | | | | | | | | |
| 3-Hydroxybutyric acid | C_4_H_8_O_3_ | 104.0473 | 8.349 |  |  | 3.5 | GC-QTOF-MS | 2 | 0.37 | 4.87E-02 | -- | -- |
| ***Indoles*** | | | | | | | | | | | | |
| Indole carbinol | C_9_H_9_NO | 147.0684 | 6.55 | 4 | M+H-H_2_O | 9.1 | LC-QTOF-MS | 2 | 1.91 | 8.47E-03 | 1.18E-02 | -- |
| Tetrahydropteroyltri-glutamate | C_24_H_34_N_8_O_12_ | 626.2296 | 11.24 | 7 | M+H-H_2_O | 9.78 | LC-QTOF-MS | 3 | 0.90 | 2.27E-04 | 6.67E-04 | -- |
| ***Lactones*** | | | | | | | | | | | | |
| Gulonic acid lactone | C_6_H_10_O_6_ | 178.0477 | 17.386 |  |  | 5.9 | GC-QTOF-MS | 2 | 0.78 | 2.67E-03 | 2.07E-02 | -- |
| Glucuronolactone | C_6_H_8_O_6_ | 176.0321 | 17.641 |  |  | 5.4 | GC-QTOF-MS | 2 | 0.94 | 2.47E-02 | -- | 1.4 |
| ***Peptides*** | | | | | | | | | | | | |
| Arg Asp Phe | C_19_H_28_N_6_O_6_ | 436.207 | 12.8 | 8 | M-H | 15.02 | LC-QTOF-MS | 3 | 0.84 | 2.63E-03 | 5.39E-03 | -- |
| Ala Lys Ala | C_12_H_24_N_4_O_4_ | 288.1798 | 3.29 | 5 | M+H-H_2_O | 5.71 | LC-QTOF-MS | 3 | 0.17 | 5.31E-09 | 1.50E-07 | -- |
| Ile Leu Arg | C_18_H_36_N_6_O_4_ | 400.2798 | 3.29 | 4 | M+H | 5.42 | LC-QTOF-MS | 2 | 0.17 | 2.12E-08 | 3.61E-07 | -- |
| His Glu Trp | C_22_H_26_N_6_O_6_ | 470.1914 | 4.38 | 2 | M+H-H_2_O | 6.82 | LC-QTOF-MS | 3 | 3.08 | 1.36E-04 | 4.29E-04 | -- |
| Ser-Ser-OH | C_12_H_14_N_2_O_8_ | 314.075 | 0.62 | 6 | M+H | 3.92 | LC-QTOF-MS | 3 | 1.14 | 3.73E-15 | 3.17E-13 | 5.30 |
| Aspartyl-threonine | C_8_H_14_N_2_O_6_ | 234.0852 | 0.63 | 0 | M-H | 5.51 | LC-QTOF-MS | 2 | 1.10 | 1.00E-06 | 7.51E-06 | -- |
| Glycyl-Hydroxyproline | C_7_H_12_N_2_O_4_ | 188.0797 | 0.63 | 1 | M-H | 4.84 | LC-QTOF-MS | 3 | 1.11 | 6.34E-07 | 5.19E-06 | -- |
| Glutamylproline | C_10_H_16_N_2_O_5_ | 244.1059 | 0.62 | 8 | M-H-H_2_O | 3.79 | LC-QTOF-MS | 2 | 1.04 | 3.21E-04 | 1.02E-03 | -- |
| ***Prostaglandins*** | | | | | | | | | | | | |
| PGF2 | C_20_H_34_O_5_ | 354.2406 | 8.89 | 2 | M-H | 9.24 | LC-QTOF-MS | 3 | 1.58 | 7.38E-05 | 3.02E-04 | -- |
| Dimethyl Prostaglandin F1a | C_22_H_40_O_5_ | 384.2876 | 13.54 | 0 | M-H-H_2_O | 6.01 | LC-QTOF-MS | 3 | 0.83 | 2.11E-02 | 3.39E-02 | -- |
| ***Pyrimidines*** | | | | | | | | | | | | |
| Thioxanthine monophosphate | C_10_H_13_N_4_O_8_PS | 380.0192 | 0.59 | 0 | M+Na | 3.59 | LC-QTOF-MS | 3 | 1.12 | 3.91E-05 | 2.37E-04 | -- |
| Deoxyuridine | C_9_H_12_N_2_O_5_ | 228.0746 | 0.62 | 9 | M-H-H_2_O | 3.82 | LC-QTOF-MS | 3 | 1.04 | 1.69E-03 | 3.60E-03 | -- |
| Dihydrouridine | C_9_H_14_N_2_O_6_ | 246.0852 | 0.64 | 1 | M+HCOOH-H | 4.86 | LC-QTOF-MS | 3 | 1.10 | 3.37E-07 | 3.49E-06 | -- |
| Butyrylphosphouridine | C_13_H_19_N_2_O_10_P | 394.0777 | 11.04 | 1 | M+Cl | 12.99 | LC-QTOF-MS | 3 | 0.69 | 3.92E-03 | 7.62E-03 | -- |
| dCTP | C_9_H_16_N_3_O_13_P_3_ | 466.9896 | 11.04 | 0 | M-H-H_2_O | 7.60 | LC-QTOF-MS | 3 | 0.68 | 8.81E-04 | 2.26E-03 | -- |
| ***Sterol Lipids*** | | | | | | | | | | | | |
| Androstanol | C_19_H_32_O | 276.2453 | 12.8 | 1 | M-H-H_2_O | 8.51 | LC-QTOF-MS | 3 | 0.84 | 1.32E-02 | 2.27E-02 | -- |
| Dihydroxycholesterol | C_27_H_46_O_3_ | 418.3447 | 14.43 | 2 | M+HCOOH-H | 18.28 | LC-QTOF-MS | 3 | 0.74 | 2.03E-04 | 7.02E-04 | -- |
| Glutaryloxy dihydroxyvitamin D3 | C_32_H_50_O_7_ | 546.3557 | 11.24 | 10 | M+Cl | 10.39 | LC-QTOF-MS | 3 | 0.86 | 5.65E-04 | 1.62E-03 | -- |
| Dihydroxycholanoic Acid | C_24_H_40_O_4_ | 392.2927 | 14.37 | 1 | M-H | 11.42 | LC-QTOF-MS | 3 | 0.81 | 2.75E-02 | 4.12E-02 | -- |
| Dihydroxycholestenoate | C_27_H_44_O_4_ | 432.324 | 15 | 2 | M-H | 9.87 | LC-QTOF-MS | 3 | 0.78 | 4.68E-02 | -- | -- |
| Cholestanetetrol | C_27_H_48_O_4_ | 436.3553 | 13.93 | 2 | M+FA-H | 13.91 | LC-QTOF-MS | 3 | 0.57 | 1.83E-06 | 1.16E-05 | -- |
| Glycoursodeoxycholic acid | C_26_H_43_NO_5_ | 449.3141 | 9.23 | 2 | M-H | 8.32 | LC-QTOF-MS | 2 | 0.51 | 3.05E-04 | 9.89E-04 | 1.16 |
| Glycochenodeoxycholic acid glucuronide | C_32_H_51_NO_11_ | 625.3462 | 8.07 | 1 | M-H | 6.41 | LC-QTOF-MS | 3 | 0.74 | 1.55E-03 | 3.41E-03 | -- |
| Chenodeoxyglycocholic acid // Glycodeoxycholate // Glycoursodeoxycholic acid | C_26_H_43_NO_5_ | 449.3141 | 9.47 | 2 | M-H | 6.20 | LC-QTOF-MS | 3 | 0.67 | 2.10E-02 | 3.39E-02 | -- |
| Chenodeoxyglycocholic acid | C_26_H_43_NO_5_ | 449.3141 | 9.23 | 4 | M+H-H_2_O | 5.4 | LC-QTOF-MS | 3 | 0.51 | 5.01E-04 | 1.18E-03 | -- |
| Sulfolithocholylglycine | C_26_H_43_NO_7_S | 513.276 | 8.8 | 2 | M-H | 5.04 | LC-QTOF-MS | 3 | 0.65 | 2.42E-03 | 5.07E-03 | -- |
| Norcholestanepentol | C_26_H_46_O_5_ | 438.3345 | 14.76 | 3 | M-H | 10.92 | LC-QTOF-MS | 3 | 0.62 | 1.26E-06 | 8.67E-06 | -- |
| Taurochenodeoxycholic acid | C_26_H_45_NO_6_S | 499.2968 | 8.15 | 2 | M-H | 9.47 | LC-QTOF-MS | 3 | 0.43 | 2.04E-04 | 7.02E-04 | -- |
| Cholestadienol benzoate | C_34_H_48_O_2_ | 488.3654 | 14.92 | 11 | M-H-H_2_O | 9.72 | LC-QTOF-MS | 3 | 0.44 | 3.52E-12 | 6.06E-10 | -- |
| Hydroxygeminivitamin D3 | C_32_H_54_O_5_ | 518.3971 | 14.58 | 2 | M-H | 8.08 | LC-QTOF-MS | 3 | 0.85 | 1.49E-02 | 2.52E-02 | -- |
| Glycochenodeoxycholic acid sulfate | C_26_H_43_NO_8_S | 529.2709 | 7.93 | 2 | M-H | 4.36 | LC-QTOF-MS | 3 | 0.70 | 1.31E-03 | 3.09E-03 | -- |
| ***Sphingolipids*** | | | | | | | | | | | | |
| Sphingosine phosphate | C_18_H_38_NO_5_P | 379.2488 | 10.45 | 5 | M+H M-H | 4.1 | LC-QTOF-MS | 3 | 0.72 | 2.45E-05 | 2.08E-04 | 1.49 |
| Sphinganine-phosphate | C_18_H_40_NO_5_P | 381.2644 | 10.75 | 2 | M-H | 8.25 | LC-QTOF-MS | 2 | 0.71 | 1.50E-03 | 3.36E-03 | -- |
| PI-Cer d36:1 | C_42_H_82_NO_11_P | 807.5626 | 14.38 | 2 | M+HCOOH-H | 5.39 | LC-QTOF-MS | 3 | 0.86 | -- | -- | 1.78 |
| C17 Sphinganine (Iso 2) | C_17_H_37_NO_2_ | 287.2824 | 9.29 | 7 | M+H | 2.24 | LC-QTOF-MS | 3 | 16.6 | 7.55E-05 | 3.04E-04 | 4.03 |
| C17 Sphinganine (Iso 1) | C_17_H_37_NO_2_ | 287.2824 | 9.53 | 5 | M+H | 2.7 | LC-QTOF-MS | 3 | 17.1 | 7.87E-05 | 3.04E-04 | 5.26 |
| NeuAcHexCer 34:1;O2 | C_51_H_94_N_2_O_16_ | 990.6603 | 11.72 | 4 | M+HCOOH-H | 7.23 | LC-QTOF-MS | 3 | 1.07 | -- | -- | 1.42 |
| Ganglioside GA2 d18:1/18:0 | C_56_H_104_N_2_O_18_ | 1092.7284 | 13.52 | 4 | M-H | 4.93 | LC-QTOF-MS | 3 | 1.04 | -- | -- | 1.8 |
| ***Purines*** | | | | | | | | | | | | |
| 9H-Purin-6-ol | C_5_H_4_N_4_O | 136.0385 | 16.497 |  |  | 8.8 | GC-QTOF-MS | 2 | 0.55 | -- | -- | 2.1 |

^a^CV, coefficient of variation in the metabolites in the QC samples; ^b^Identification level: Level 1 Structure confirmed, Level 2 Structure probable, Level 3 Unequivocal molecular formula (s), Level 4 Exact mass; ^c^Fold Change, change in the abundance of the specified comparison calculated as (case/control); LC: liquid chromatography, GC: gas chromatography, QTOF-MS: quadrupole time-of-flight mass spectrometer; RT: retention time.


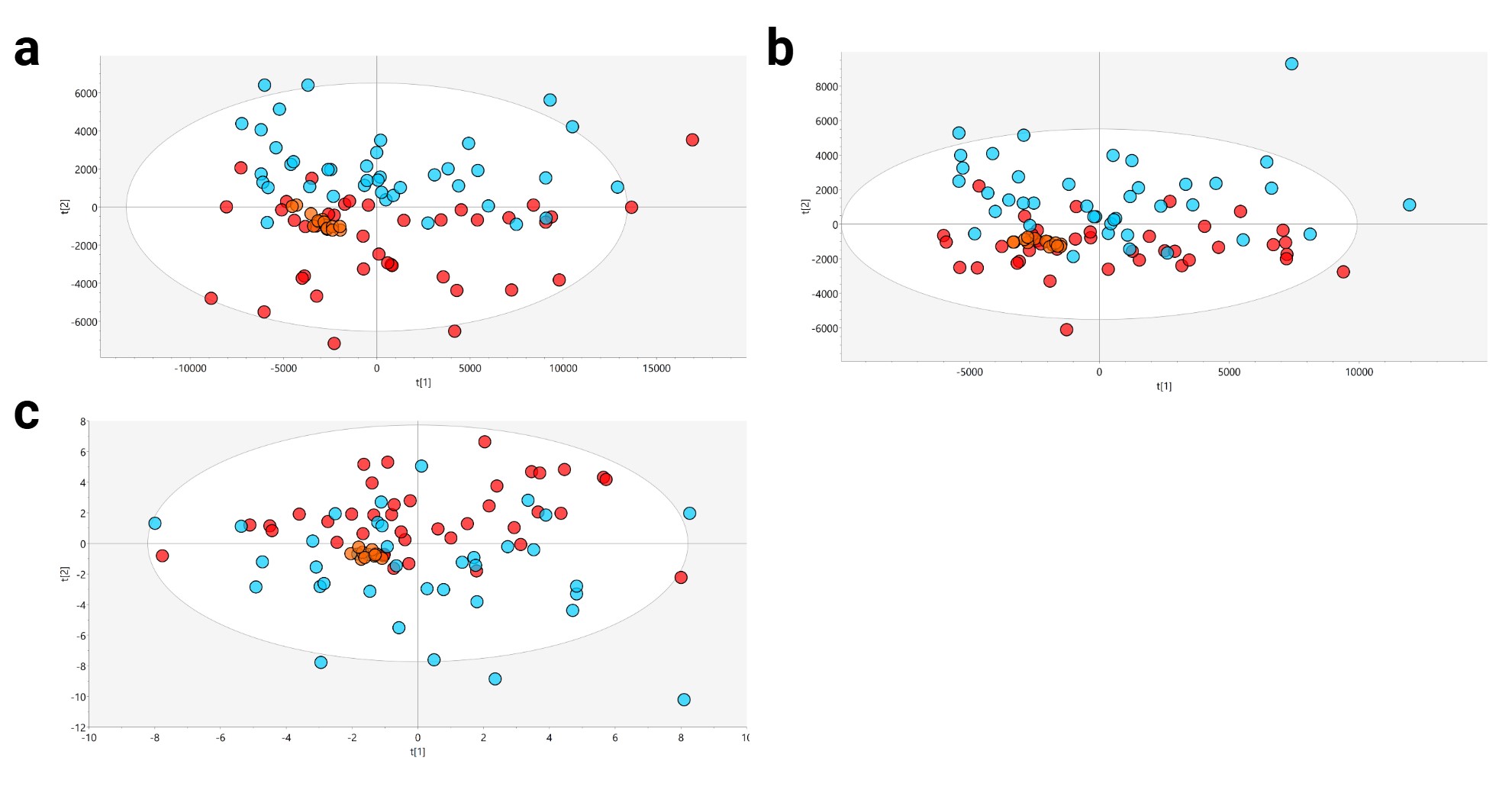


**Figure S1.** PCA Models. a) LC-MS(+): R^2^Y: 0.856. b) LC-MS(-): R^2^Y: 0.834. c) GC-MS: R^2^Y: 0.828. Dots in red, blue and orange colors denote samples from altitude natives, the lowlanders group and QC, respectively.


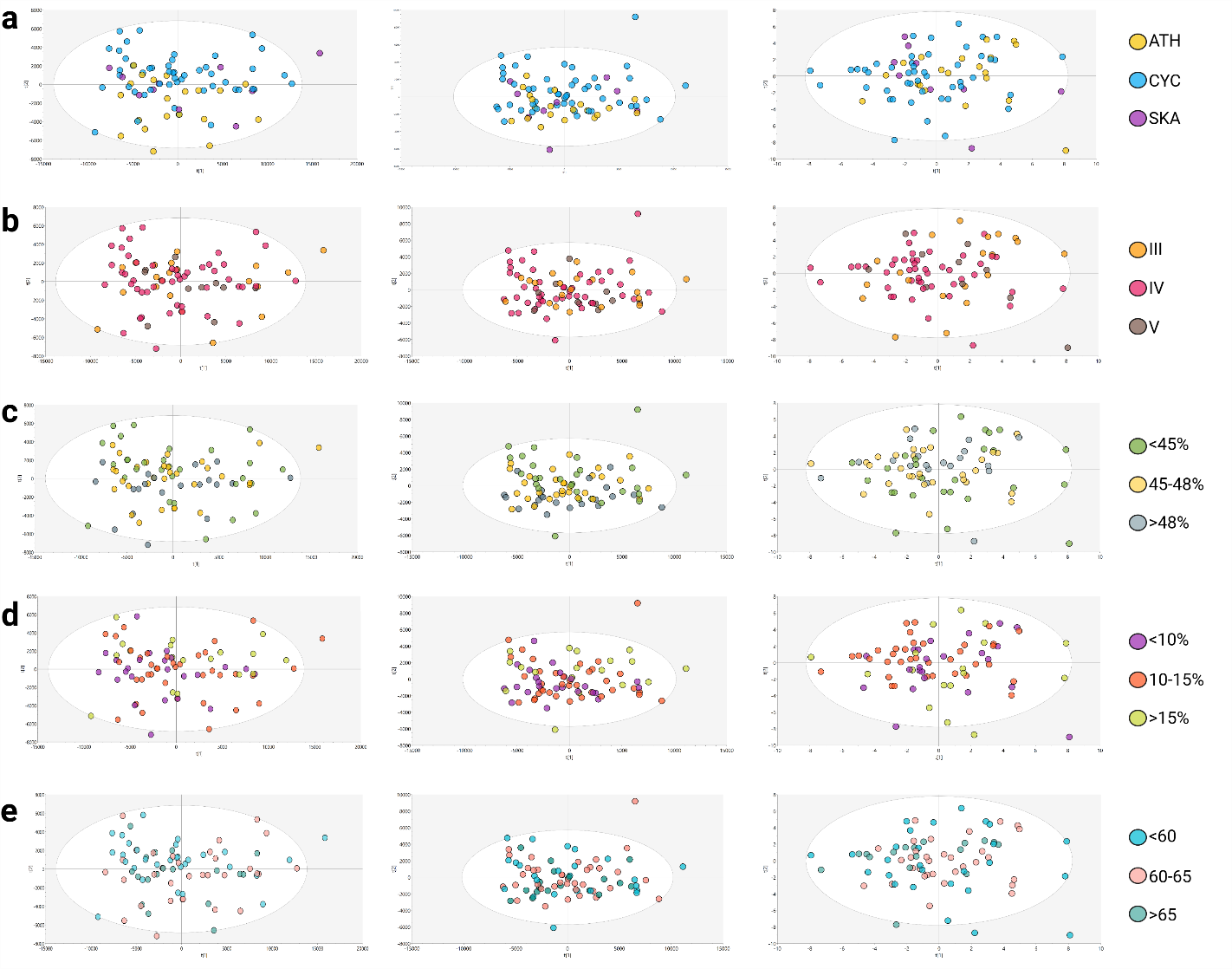
 **Figure S2.** Exploratory Principal Component Analysis (PCA) of individual characteristics. (A) Type of sport practiced—athletics (ATH), cycling (CYC), or inline skating (SKA); (B) Biological maturation status based on Tanner stages (III, IV, and V); (C) Hematocrit (Htc%) categorized as <45%, 45–48%, and >48%; (D) Percent body fat (%) classified as <10%, 10–15%, and >15%; (E) VO₂peak (mL∙min⁻¹∙kg⁻¹) categorized as <60, 60–65, and >65. The classification of samples is represented according to the figure legend.


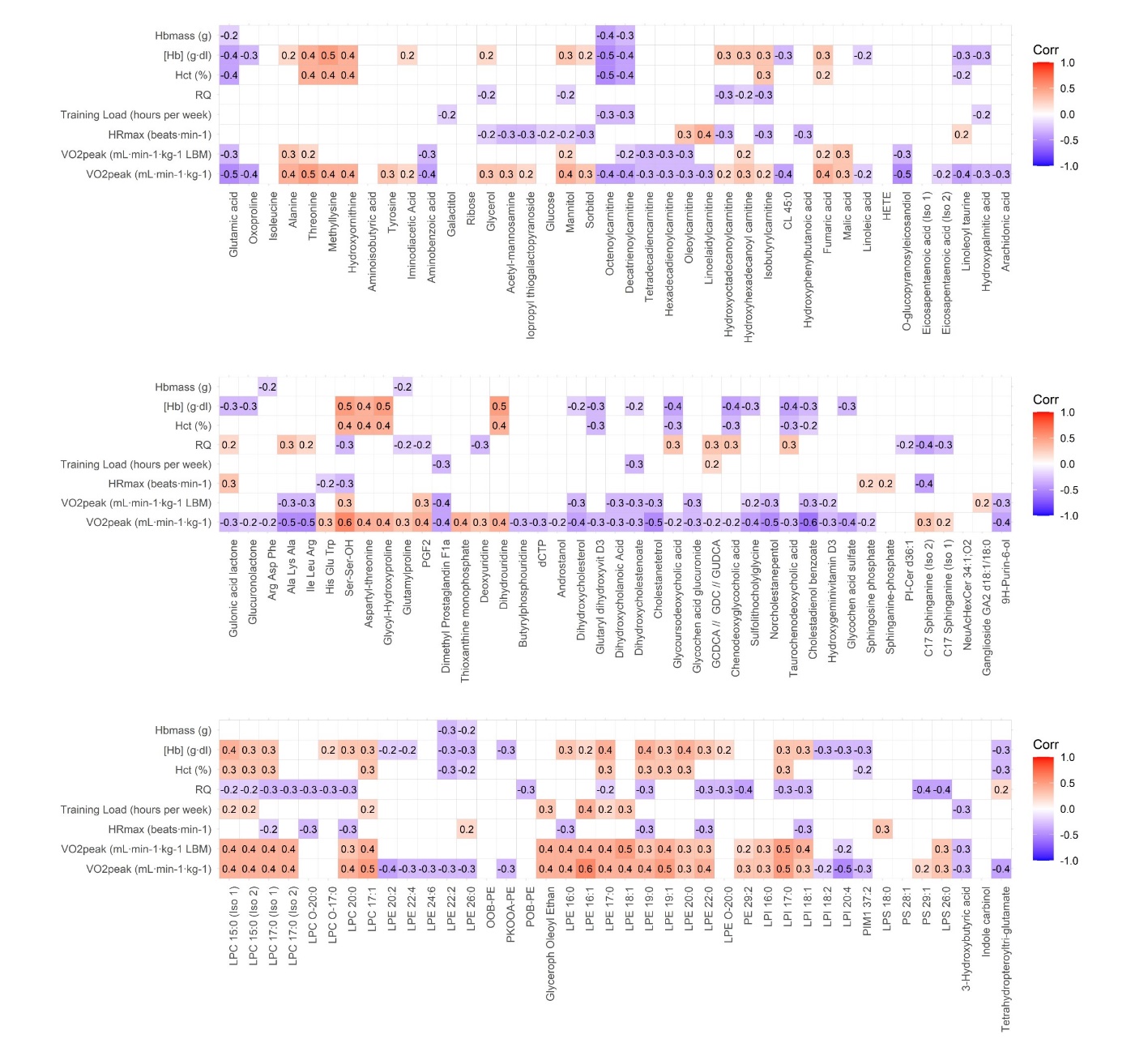
**Figure S3.** Metabolites correlation with physiological and hematology data. The degree of correlation is presented according to the color scale, where red colors indicate positive correlations and purple colors indicate negative correlations.

# References

HERMANSEN, L. & SALTIN, B. 1969. Oxygen uptake during maximal treadmill and bicycle exercise. *Journal of applied physiology,* 26**,** 31-37.

HOWLEY, E. T., BASSETT, D. R. & WELCH, H. G. 1995. Criteria for maximal oxygen uptake: review and commentary. *Medicine & Science in Sports & Exercise,* 27**,** 1292-1301.

M-JM, S. A., OLDS, T. & DE RIDDER, H. 2011. International Standards for Anthropometric Assessment. *Lower Hutt, New Zealand: ISAK*.

MANCERA-SOTO, E. M., CHAMORRO-ACOSTA, M. L., RAMOS-CABALLERO, D. M., TORRELLA, J. R. & CRISTANCHO-MEJÍA, E. 2022. Effect of hypobaric hypoxia on hematological parameters related to oxygen transport, blood volume and oxygen consumption in adolescent endurance-training athletes. *Journal of Exercise Science & Fitness,* 20**,** 391-399.

SLAUGHTER, M. H., LOHMAN, T., BOILEAU, R., HORSWILL, C., STILLMAN, R., VAN LOAN, M. & BEMBEN, D. 1988. Skinfold equations for estimation of body fatness in children and youth. *Human biology***,** 709-723.
